# Plant Growth Environments with Programmable Relative Humidity and Homogeneous Nutrient Availability

**DOI:** 10.1371/journal.pone.0155960

**Published:** 2016-06-15

**Authors:** Kara R. Lind, Nigel Lee, Tom Sizmur, Oskar Siemianowski, Shawn Van Bruggen, Baskar Ganapathysubramaniam, Ludovico Cademartiri

**Affiliations:** 1 Department of Materials Science & Engineering, Iowa State University of Science and Technology, Ames, Iowa, United States of America; 2 Department of Mechanical Engineering, Iowa State University of Science and Technology, Ames, Iowa, United States of America; 3 Department of Geography and Environmental Science, The University of Reading, Reading, United Kingdom; 4 Department of Chemical & Biological Engineering, Iowa State University of Science and Technology, Ames, Iowa, United States of America; 5 Ames Laboratory, U.S. Department of Energy, Ames, Iowa, United States of America; United States Department of Agriculture, Agricultural Research Service, UNITED STATES

## Abstract

We describe the design, characterization, and use of “programmable”, sterile growth environments for individual (or small sets of) plants. The specific relative humidities and nutrient availability experienced by the plant is established (RH between 15% and 95%; nutrient concentration as desired) during the setup of the growth environment, which takes about 5 minutes and <1$ in disposable cost. These systems maintain these environmental parameters constant for at least 14 days with minimal intervention (one minute every two days). The design is composed entirely of off-the-shelf components (e.g., LEGO^®^ bricks) and is characterized by (i) a separation of root and shoot environment (which is physiologically relevant and facilitates imposing specific conditions on the root system, e.g., darkness), (ii) the development of the root system on a flat surface, where the root enjoys constant contact with nutrient solution and air, (iii) a compatibility with root phenotyping. We demonstrate phenotyping by characterizing root systems of *Brassica rapa* plants growing in different relative humidities (55%, 75%, and 95%). While most phenotypes were found to be sensitive to these environmental changes, a phenotype tightly associated with root system topology–the size distribution of the areas encircled by roots–appeared to be remarkably and counterintuitively insensitive to humidity changes. These setups combine many of the advantages of hydroponics conditions (e.g., root phenotyping, complete control over nutrient composition, scalability) and soil conditions (e.g., aeration of roots, shading of roots), while being comparable in cost and setup time to Magenta^®^ boxes.

## Introduction

We are interested in understanding the role of environmental factors in the development of plants and ecosystems. Our initial effort focuses on developing laboratory scale growth environments that control and monitor the environment of individual plants in space and time (e.g., humidity, water availability, nutrient availability) during their growth. This capability is currently not possible in the field and is beyond the common protocols and infrastructures of laboratories (e.g., growth chambers).

We describe in this paper an experimental system that provides self-contained, sterile, growth environments for individual plants that are programmable to control (for at least 14 days) constant relative humidity (RH, between 15% and 95%) and homogenous nutrient availability. In these environments, the root system develops onto a flat sheet of paper that is saturated with the nutrient solution. The seed is sowed into a plug that is lodged into a plastic sheet that separates the environment of the root from that of the shoot. The separation between the root and the shoot environment is important because (i) it reduces the evaporation from the nutrient reservoir, which eliminates nutrient accumulation and enables an effective control of humidity at the shoot, (ii) it facilitates the shading of the root system from light (cf. S10 Fig), and (iii) it is more similar to the physiological growth conditions of the plant. An earlier design of this approach achieved a homeostatic control of humidity through the use of saturated salt solutions, but could not limit the accumulation of nutrients in contact with the roots due to evaporation of the nutrient solution[1]. Furthermore, the range of attainable relative humidities was limited between ~50% and 95% and therefore could not simulate truly desiccating conditions.

Growth chambers or phytotrons for individual (or few) plants provide several advantages over larger scale equipment (e.g., large growth chambers) or facilities (e.g., greenhouses). *Environmental control*. Because of the historical emphasis on studying and breeding plants in loosely defined “physiological” environments, the current infrastructure and methods for plant science and breeding are very sophisticated when it comes to plant characterization (e.g., confocal microscope, Genome-wide association studies), but less so when it comes to plant growth. Humidity, for example is a very difficult parameter to control, especially at scale [2–5]. Other parameters (e.g., nutrient composition, heterogeneities such as nutrient gradients) are difficult to control in time and space (especially in field trials) since they are dependent on the type of "soil" media the plants are growing in [6, 7]. Controlling environments is easier in small volumes than it is in large volumes (think, for example, about sterile conditions): our environments maintain constant humidity and nutrient concentration in sterile conditions without requiring electrical power. *New data*. Standardized, self-contained, highly modular, and customizable plant environments enable unique experiments based on exposing plants to unique environmental stimuli. Many of the most interesting questions with respect to plant development relate to how local environmental cues lead to a global phenotype. *Individual stress testing*. Due to the ineffectiveness of growth chamber/greenhouse environments at testing plants' responses to the environment, the bulk of the "stress-testing" of plants in breeding is performed in field trials. These pipelines are expensive and slow and have a low success rate [8, 9] also because stress intolerant plants were not removed from the candidate pool at the greenhouse stage. It is therefore useful to develop systems that grow individual (or small groups of plants) plants with a better control of environmental conditions for laboratory scale experiments as well as large phenotyping trials. Individual plant environments would allow stress testing on a select number of plants in laboratories. *Logistics*. Individual, self-contained growth environments would enable the plant science experiments without requiring dedicated, expensive growth facilities (rhizotrons, growth chambers, greenhouses) that may not be available to researchers from other disciplines. *Reproducibility*. The lack of universally embraced standards in plant growth protocols considerably reduces reproducibility[10]. Despite internal controls, many environmental variables are almost never rigorously controlled for (e.g., biotic environment of plants, light quality). The development of integrated, standardized tools for controlling the environment surrounding individual plants would enable improvements in experimental reproducibility that are necessary to address complex biological questions such as Genome-by-Environment (GxE) effects. *Failure tolerance*. Single plant environments, because they are confined and distributed, limit and contain failure (e.g. due to disease or contamination), thereby reducing the risk of catastrophic experiment loss. *Robustness*. Because of ther untethered, simple design, single plant environments are less likely to break, to malfunction, to degrade. *Higher data quality*. Single plant chambers with accurate environmental control could reduce experimental variability and therefore enable the design of experiments that reduce replicate numbers in favor of highly controlled environmental conditions with low failure rates. Data quality and highly controlled experiments is an approach to bring value to small laboratory operations to complement large facilities.

The plant/soil/environment system is a complex, highly correlated system. There are two main approaches to studying such systems: a holistic approach, preferably data-intensive, in which the real system is monitored in its full complexity and where analysis of the data can bring out correlations, suggest hypotheses, and sometimes make predictions [11, 12]. The other is a reductionist approach that produces model systems in which a select number of variables (typically very few) can be independently changed and monitored, therefore enabling the systematic testing of hypotheses[12, 13].

The first approach is increasingly common in plant science, as shown by the use of sophisticated characterization techniques for phenotyping in facilities [14–18] and in the field [19, 20], with the intent to produce higher quality and quantity of data for predictive phenotyping. The second approach is also very common in plant science but is mostly focused on *organismal* model systems (e.g., Arabidopsis thaliana, Populus trichocarpa) rather than *environmental* model systems (e.g., Petri dishes, Magenta boxes, phytotrons), which have not substantially improved over the past decade. While these very simple environmental model systems have been invaluable in developing knowledge, and useful in formulating and rapidly testing hypotheses [21–24] they do not provide a close enough model of field conditions (leading, for example, to a frustrating lack of correlation between lab performance and field performance of plants), and they cannot adequately provide reproducibility across labs and field conditions [10, 25]. With the help of the engineering toolbox, environmental model systems can be designed to rigorously, robustly control previously challenging or inaccessible environmental variables (e.g., chemical gradients, microbiome), while remaining simple, cheap, scalable, reusable, modular, and easy to use [1, 26].

## Experimental Design

Plants are systems out of equilibrium which drive change in their environment by moving mass and energy and reacting chemicals. Therefore, it is challenging to create simple systems that establish a programmed steady state and that, at the same time, fulfill a long list of design constraints associated with experimental plant science. For a growth environment to be useful for plant studies it should be scalable (and therefore inexpensive and untethered from electrical power), simple to assemble, chemically inert, autoclavable, transparent, and relying on off-the-shelf components.

We wish our systems to be operated outside of sterile environments, e.g., on a laboratory benchtop. Therefore we opted for a fully enclosed system that can be easily and rapidly (5 min) assembled in a biosafety cabinet (cf. S1 Movie) and then placed anywhere. The outside enclosure should be transparent for illumination and we used a commercially available polypropylene box (Sterilite^®^ brand).

**Fig 1:**
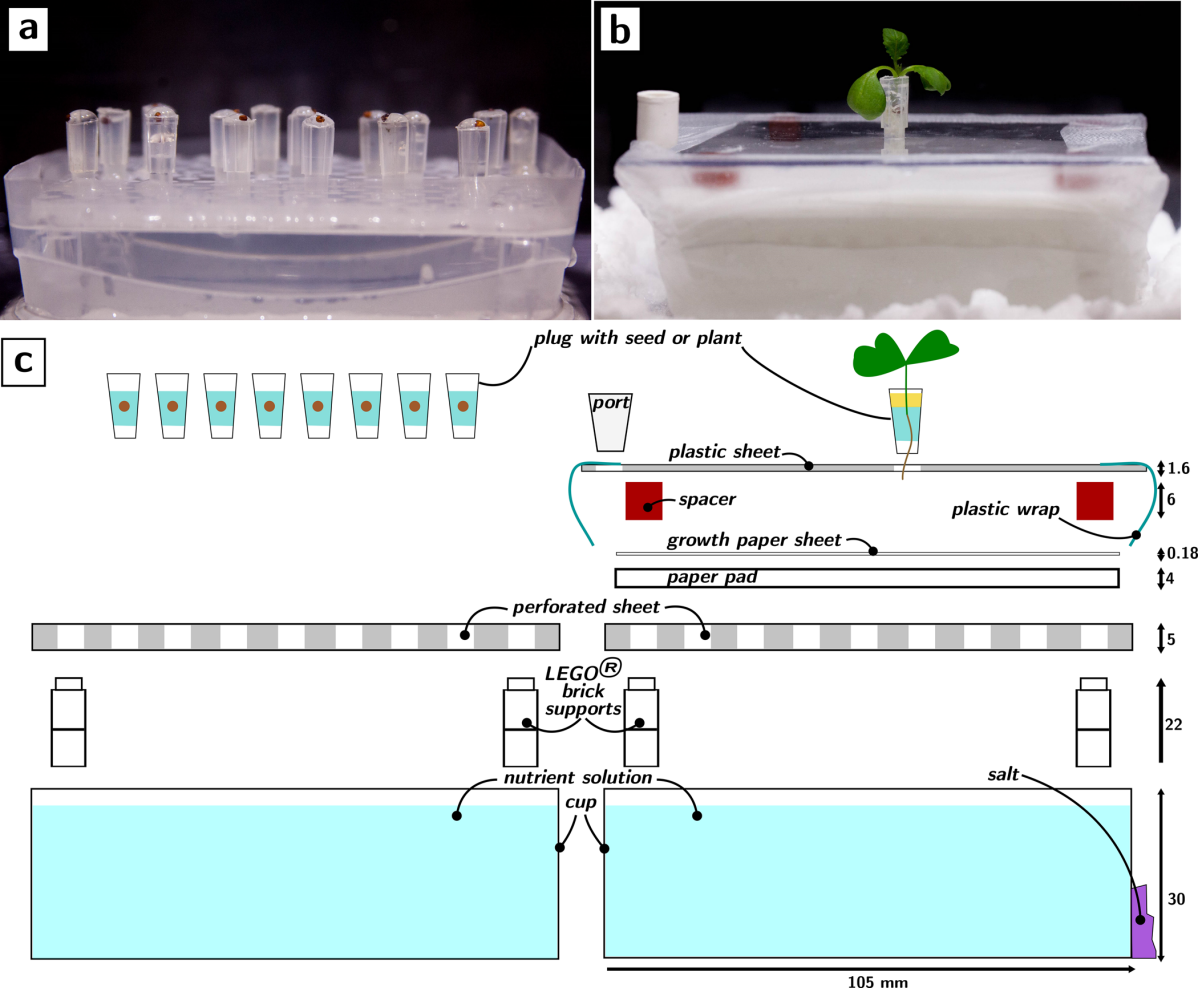
Germination and growth environments. Pictures and exploded views (external enclosures omitted for clarity). a) Side view of the germination setup. b) Side view of the growth setup with a *Brassica rapa* plant. c) Exploded views, to scale, of the germination (left) and growth (right) environments (units of length are mm).

Separate, dedicated, germination environments are useful because they allow to select similarly developed plants as replicates for experiments in the growth environments. We desired our germination environment to be as similar as possible (so as not to require an unnecessary number of different parts) and that would allow us to transfer the germinated seeds to the growth environment in a rapid (<1min) and simple manner (cf. S2 Movie). The germination and growth environments are shown in Fig 1A and 1B, respectively (the outer enclosure is omitted for clarity). Corresponding exploded views of the setups are shown in Fig 1C, highlighting the similarities between the two setups.

In the germination environment (Fig 1A), a plastic cup is used to hold nutrient solution. A perforated plastic sheet is suspended horizontally in the nutrient solution with the help of transparent (i.e., polycarbonate) LEGO^®^ bricks. Seeds of the plant to be germinated are sowed into a gel (0.5% agar) held by pipette tips, which are then lodged into the perforations of the plastic sheet until their bottoms dip into the nutrient solution. The seeds germinate in the plug and the roots grow out of the holes at the bottom into the hydroponic solution. This hydroponic geometry greatly simplifies the handling of large numbers of seeds and the maintenance of the system. The use of plugs (i.e., cut pipette tips) to hold the seeds enables the rapid transplantation of the germinated seedling to the growth environment.

The growth environment differs from the germination environment only by a few components. A pad of paper (Whatman #1 filter paper or blotting paper) is placed above the perforated plastic sheet and is nearly fully immersed in the nutrient solution. On top of the pad is a single sheet of paper (the “growth” sheet, Whatman #1 filter paper). The growth sheet wicks water and nutrients from the saturated paper pad. On the four corners of the growth sheet are four silicone rubber spacers that support a polycarbonate sheet with a hole in its middle. The seed plug started in the germination environment is placed in this hole. The top plastic sheet is fitted with a port for drawing and introducing liquids into the nutrient cup and the whole system is wrapped by plastic wrap. This closed environment is then placed into the outer enclosure surrounded by salt that establish the desired humidity in the environment of the shoot.

The setups are entirely reusable, with the exception of the paper pad and growth sheet. The salt can be dried in a rotary evaporator or an oven. The cost of the setup shown is <8$, while the cost per experiment is <1$ even with the cost of the seed. The setup can be easily scaled and its capabilities are conserved as long as these essential characteristics are preserved: (i) a short distance (<3 mm) between the level of the nutrient solution and the growth sheet, (ii) a paper pad with a thickness equal or greater than the typical separation between the holes in the perforated sheet, (iii) a proper seal of the nutrient cup with plastic wrap (or analogous method) to limit evaporation of the nutrient solution, (iv) a port to replenish the nutrient cup as necessary.

The seedlings transplanted from the germination environment develop their roots onto the growth sheet, remaining in constant contact with both their nutrient and water supply as well as air. This approach allows to us combine the advantages of hydroponics (e.g., tight control over nutrient availability) and particulate systems (e.g., root access to oxygen) at the expense of the three-dimensionality of the root system. 2D root systems are very common in the study of roots by the use of rhizotrons or rhizoslides. The main differences between our approach and rhizotrons are that the growth sheet in this system is held horizontal, and that the roots are exposed to air. As it will be shown later, growth on flat surface tends to produce a more entangled but also more symmetric root system that could facilitate the detection of weak tropisms and root development responses.

## Results and Discussion

**Fig 2:**
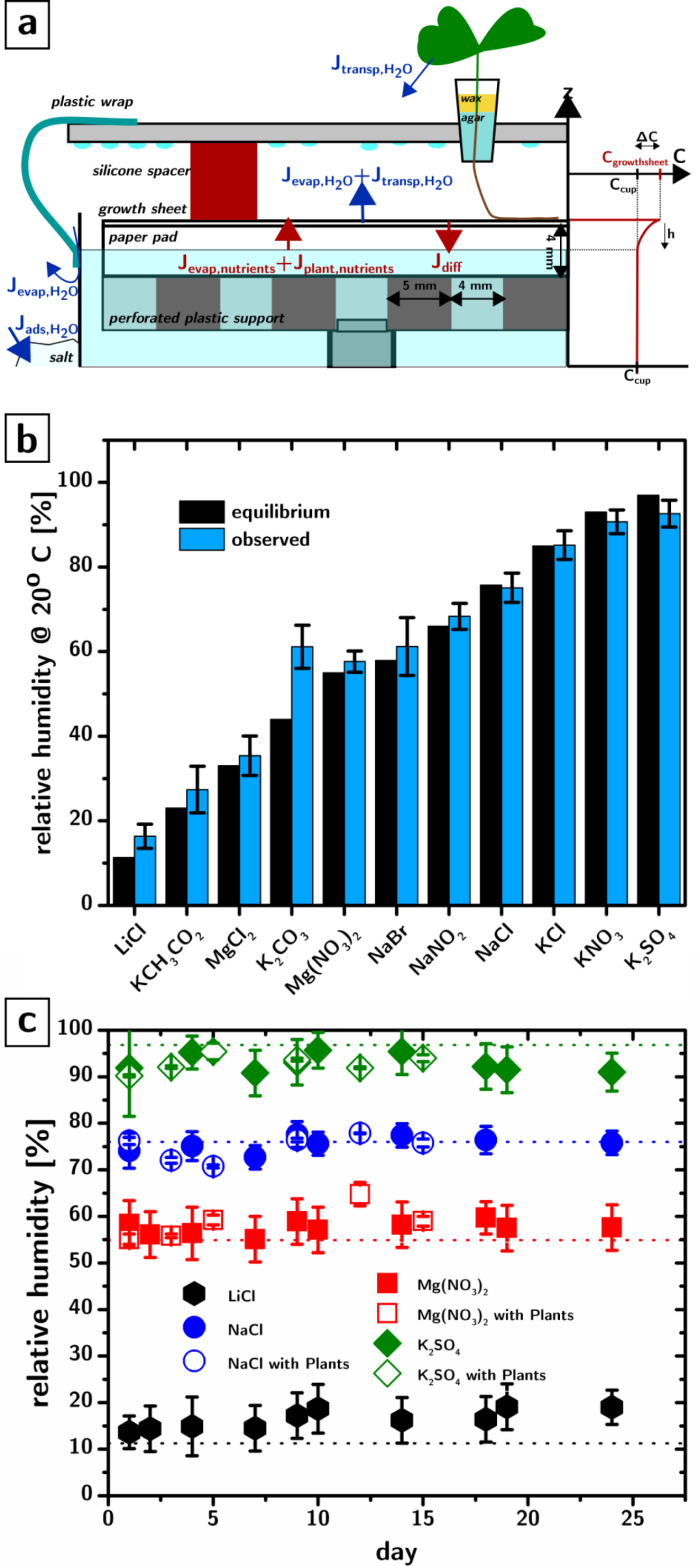
Mass flows in the growth environment and humidity control. a) Schematic of the water flows (blue arrows) and nutrient flows (red arrows) in the growth environment. On the side is a depiction of the nutrient concentration gradient formed in the part of the paper support that is exposed to evaporation. b) Observed relative humidities measured in the shoot environment, compared to the equilibrium values for a number of different supersaturated salt solutions. Error bars are 95% confidence intervals, n = 2. c) Observed relative humidities as a function of time for systems without (filled symbols) and with a plant of *Brassica rapa* plants (open symbols), compared to the equilibrium values at 20°C (dotted lines). Error bars are 95% confidence intervals, n = 15.

Establishment of a programmed steady state of nutrient concentrations and humidities requires an understanding of the mass flows into the system caused by evaporation and transpiration (Fig 2A).

The water cycle in the system is fairly simple. Water from the nutrient cup is wicked by the paper pad and the growth sheet from which it evaporates into the root environment. Since the root environment is a closed system the humidity reaches rapidly 100%, leading to condensation. Some leaks lead to a net water loss from the root environment into the shoot environment through evaporation (J_evap_). As it will be shown, the design tolerates minor leaks without compromising the control over RH and nutrient concentrations. Evaporation of the agar in the seed plug is prevented by sealing the agar in the plug with wax (this step is essential to prevent the drying of the agar in the first day after transplantation). Water is also extracted from the nutrient cup by the root system and the majority of it is then transpired by the leaves in the shoot environment (J_transp_) while the remainder (usually less than 1%[27]) is stored in plant tissues. The shoot environment is a closed environment: in the absence of water sinks, the humidity reaches rapidly 100%. In our setup, hygroscopic salt (e.g., NaCl) is added on the outside of the nutrient cup and acts as a water sink. The adsorption of the water by the salt (J_ads_) will, at steady state, match the combined flow of water from evaporation and transpiration (J_evap,H2O_ + J_transp,H2O_), and establish a steady state RH. The value of the RH at steady state will depend on the composition of the salt (any supersaturated solution establishes a certain vapor pressure of water at equilibrium[28]) and on kinetics. If the rate at which water vapor is introduced in the shoot environment is larger than the maximum rate at which the salt can absorb it (which will depend, in first approximation, on the area of the exposed supersaturated solution), then the average relative humidity established at steady state will be larger than the one predicted by equilibrium thermodynamics in a closed system. These kinetic limitations were the key issue with the previous design in which the growth sheet was exposed to the shoot environment, therefore yielding a very large J_evap,H2O_, especially for low humidities: LiCl, which establishes a RH of ~11% at room temperature at equilibrium was only able to reduce the humidity of the environment to ~50%. The homeostatic regulation of RH, of course, persists only as long as the salt forms a supersaturated solution. After the salt has completely dissolved, the RH will gradually increase.

The steady state rate of water loss from the nutrient cup will be J_evap,H2O_ + J_transp,H2O_. This rate will be matched exactly by J_ads,H2O_ leading to a constant concentration of water vapor in the shoot environment and a constant RH.

In our system the total water loss from the nutrient cup into the shoot environment (J_evap,H2O_ + J_transp,H2O_ = 4+1 ml/day) was low enough that the RH in the shoot environment (measured through port 2 cm above height of plastic sheet) is close to the equilibrium value (from ~15% with LiCl to ~95% with Na_2_SO_4_). Fig 2B shows the observed RH (n = 2) in the shoot environment (blue) as a function of the salt used, compared to the expected equilibrium RH (black). The small discrepancy between observed and equilibrium values is consistent with minor leaks in the external enclosure and with the exchange of water vapour with the laboratory environment, whose humidity is generally around 50%. Since our systems provide a sterile environment over at least 3 weeks, we attribute the leaks to the specific (and apparently imperfect) modifications (a ~3 cm hole in the top) we had to implement on the external enclosure to fit a hygrometer.

The programmed steady state RH was preserved for over three weeks (Fig 2C) and was maintained even in the presence of a plant for at least two weeks (*Brassica rapa*’s root system would outgrow the system after that). The data in Fig 2C show the RH observed (n = 15) in the shoot environments as a function of time and salt, with (open symbols) and without (filled symbols) a plant. We were not successful in transplanting a plant into the 15% humidity environment produced by LiCl probably due to severe transpiration stress added onto the transplantation shock. Methods for changing the RH over time will be the subject of future work. Future work will also provide control over other environmental parameters such as temperature and aeration which cannot be currently controlled independently from relative humidity and nutrient concentration. With the current design, temperatures inside the system are usually one degree Celsius above the ambient room temperature and aeration relies on the diffusion of CO_2_ through the Parafilm seal in the outer container, which is, at the moment insufficientg to maintain stationary CO_2_ levels for mature plants.

The transport of nutrients is connected with the transport of water and adsorption to surfaces. As water evaporates from the root environment, nutrients concentrate on the growth sheet (at a rate J_evap,nutrients_ = J_evap,H2O_*[C]*FW/0.01, where [C] is the molarity of the nutrient in mol/l, FW is the formula weight in g/mol). Transpiration also drives nutrients to the growth sheet (J_transp,nutrients_), some of which will be absorbed by the plant (J_ads,nutrients_). Accumulation of nutrients on the growth sheet due to water transport in the system will establish a gradient of concentration of nutrients which will drive a flow of nutrients (J_diff_) from the growth sheet back into the bulk nutrient solution. We can overestimate the expected accumulation of nutrients at the growth sheet by making the following assumptions. We approximate that the concentration of nutrients throughout the bulk of the nutrient solution is constant (C_cup_). The distance between the surface of the nutrient solution and the surface of the growth sheet, *h*, is typically 1mm but can be overestimated at 2mm. We neglect J_ads,nutrients_, thereby assuming that all nutrients brought to the growth sheet by J_evap,H2O_ + J_transp,H2O_ accumulate on the growth sheet. In our experiments J_evap,H2O_ + J_transp,H2O_ = 0.05 ml/cm^2^·day, which, for phosphate, corresponds to J_evap,nutrients_ + J_transp,nutrients_ = .001 mg/cm^2^·day. At steady state, this flow of nutrients is matched by the downward flow of nutrients (J_diff_) driven by the difference ΔC in the concentration of phosphate between the top of the growth sheet C_growthsheet_ and the nutrient cup C_cup_. Using a value of diffusivity of 0.89 x 10−^5^ cm^2^/s [29] and solving J_diff_ = D·ΔC/h for ΔC gives an estimated steady state concentration of nutrients at the growth sheet that is only 1.3% higher than that in the nutrient cup. Nutrients can also adsorb onto surfaces and become unavailable to the plant. In our system the nutrient solution contains a rather large amount of paper that can coordinate ions. It is important to compare the concentration of nutrients in the bulk liquid and compare it to the concentration introduced into the system.

**Fig 3:**
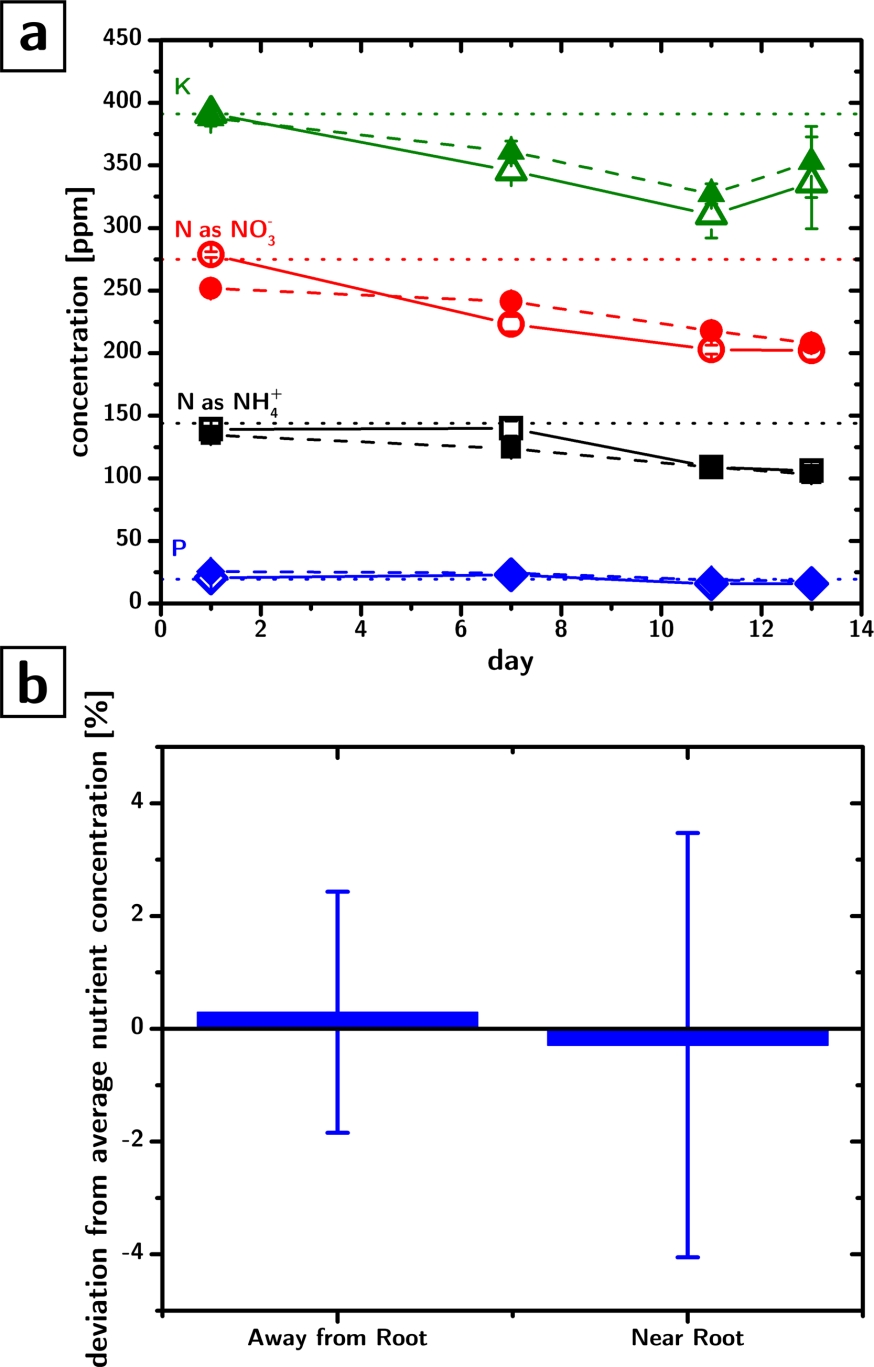
Nutrient concentrations in the growth environments. a) Concentrations of essential nutrients measured in the nutrient cup (filled symbols) and on the growth sheet (open symbols), as a function of time, in the presence of growing *Brassica rapa* plants (error bars are 95% confidence intervals, n = 48). Dotted lines indicate the initial concentration of nutrients (0.5 Murashige and Skoog, MS) b) Deviation from average nutrient concentration in regions proximal to the root (<5 mm), and away from the root (>5 mm), (error bars are 95% confidence intervals, n = 24)

Fig 3A shows the concentration of essential nutrients in the nutrient cup (open symbols and dashed lines, n = 8) as well as on the growth sheet (filled symbols, n = 8) during the growth of a plant (*Brassica rapa*) for about two weeks.

The data indicates that (i) there is no nutrient accumulation for about 2 weeks of plant growth (the concentrations in the cup are not significantly different from those observed on the growth sheet), and that (ii) the large paper pad does not immobilize a significant fraction of the nutrients in the nutrient solution. The moderate decrease in the nutrient concentration can be attributed to plant uptake, since the liquid level in the nutrient cup was always reestablished with DI water (i.e., there was no input of nutrients in the system throughout the experiment).

The flow of nutrients in the system is not only limited to the vertical axis but also occurs horizontally. Any heterogeneity in the horizontal distribution of nutrients on the growth sheet would result in an uneven distribution of nutrients across the root system of the plant, thereby driving chemotropic root development. The overall point to point concentration heterogeneity (one standard deviation) in our system was 11%. Fig 3B shows the average deviation from the average growth sheet nutrient concentration of the points of the growth sheet located close to the roots (<5mm) versus those located far from it (>5 mm), and shows that there is no significant difference between the two (p = 0.83). This data indicates that the adsorption of nutrients from the growth sheet does not lead to a significant nutrient depletion or accumulation in proximity of the root. The result is meaningful especially when comparing it with the nutrient depletion observed around the root systems grown on gels and other media[30].

**Fig 4:**
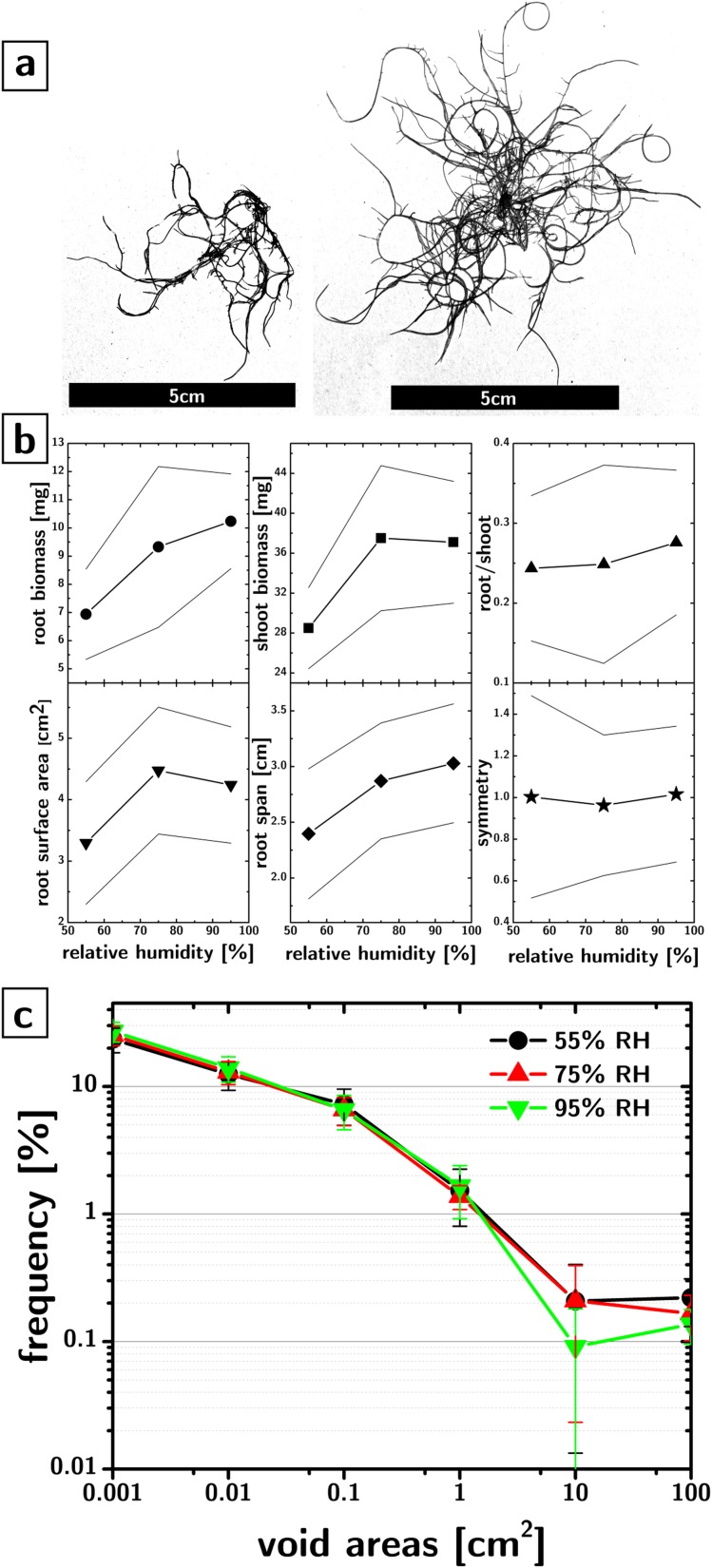
Root phenotyping. a) Representative thresholded images of root systems of *Brassica rapa* grown in 55% RH (left) and 95% RH (right). b) phenotypes as a function of RH (55%, n = 15; 75%, n = 19, 95%, n = 17): root biomass (circles), shoot biomass (squares), and root/shoot biomass ratio (up triangles) as compared to the surface area (down triangles), the span (rhombi), and the symmetry (stars) of the root system. The lines between scatters are guides to the eye. The lines above and below the scatters identify 95% confidence intervals. c) Frequency of the sizes of areas on the growth paper that were fully enclosed by roots of *Brassica rapa* plants grown in 55%, 75%, and 95% RH.

The platform is compatible with root phenotyping, albeit not *in situ*. The stem must be severed to expose the root system. Fig 4A shows the comparison of the root systems of two *Brassica rapa* plants grown in 95% and 55% RH, respectively.

The biomass of the root and shoot (Fig 4B) depends on the humidity experienced by the shoot (p = 0.02 and p = 0.03 for a significant difference between 55% and 95% RH for root and shoot biomass respectively), while the ratio between the biomass of the root and shoot did not change significantly. The biomass information is closely correlated to root phenotypes obtained through image analysis of photographs of the root system, e.g., root surface area, root span (calculated as half of the maximum width of the root system). For example, the Pearson product-moment correlation coefficient between root span and root biomass is 0.9996, while it is 0.96 between root span and shoot biomass. This finding suggests that simple analysis of root system photographs can yield–with prior calibration–biomass information even for highly overlapped root systems grown on a flat surface.

The ratio between maximum perpendicular dimensions of the root system (“root symmetry” phenotype, Fig 4B) indicates that the root system is highly symmetric in our growth environments, thereby supporting the possibility of studying quantitatively weak tropisms by quantifying asymmetry of the root system.

Root systems are generally considered to be extremely plastic to their environment[31]. While phenotypes that strongly respond to environmental conditions are useful for studying and optimizing GxE interactions, phenotypes that are robust towards environmental parameters (albeit rare) can be also useful in assessing phenotypic changes induced purely by the genotype. Fig 4C shows a root architecture phenotype that displays a remarkable robustness against relative humidity changes. Analysis of the thresholded root photographs allowed us to extract the areas (in cm^2^) that were fully enclosed by roots. The distribution of these areas is shown in Fig 4C for all sets of plants, in a log-log plot. The coincidence between the distributions is very striking, especially considering that the roots had to be transferred to a black support before their imaging, and that the thresholding process was not flawless (e.g., the distribution is likely truncated at large areas because their large perimeters make them especially subject to imperfect thresholding). The relatively linear trend on a log-log plot indicates the possibility that the void areas follow a power-law scaling that is characteristic of self-similar and fractal structures[32].

## Conclusions

We have shown a practical approach to the germination and growth of seedlings in nearly homeostatic conditions of relative humidity (between 15% and 95%) and nutrient concentrations. The setups are completely self-contained, untethered, and create two separate environments for the root and for the shoot. The root system develops on a moist, flat sheet of paper, in ~100% RH, but in constant contact with air. The shoot develops in an environment whose humidity is determined by a supersaturated salt solution. The initial conditions of their assembly are used to program the RH and nutrient concentrations that the plant will experience for 2–3 weeks. The nutrient concentrations are found to not change substantially over the course of two weeks, with minimal spatial variations, regardless of the proximity of a plant root.

The general design can be easily scaled to larger plants and can be modified to allow for different environmental conditions (e.g., shading of the root). The specific setups reported here cost <8$ (the cost per experiment is <1$ including the cost of the seed), and can be assembled in 5 min.

## Supporting Information

S1 Movie Assembly of experimental system. (MOV)

S2 Movie Assembly of germination system. (MOV)

S1 Data (XLSX)

S1 Methods (DOCX)

S1 Fig Nutrient cup before and after cutting walls of container. (TIF)

S2 Fig Fabricating perforated plastic support. a.) cut perforated polypropylene sheeting b.) rounding corners using paper cutter c.) finished perforated sheeting for platform
(TIF)

S3 Fig Polycarbonate plastic sheeting amendments. a.) Traced out size for platform b.) Clamp sheet to table with metal straight edge and score plastic with utility knife c.) Snap plastic into two pieces d-f.) Septum attached to plastic sheet g.) Finished polycarbonate plastic sheeting for platform
(TIF)

S4 Fig Modifications to external container. a.) 1” hole drilled into external container lid b.) Placement of port
(TIF)

S5 Fig Preparation of plug and spacers. a.)Before cutting and after cutting pipette tip for agar plug b.) Silicone spacers
(TIF)

S6 Fig Preparing system for sterilization. a.) Assembly of perforated LEGO^®^ support b.) Finished LEGO^®^ support c.) Preparing system for autoclaving d.) System ready for sterilization
(TIF)

S7 Fig Agar plug assembly for multiple systems intended to study germination and growth. a-e.) Sterilized brassica seeds are placed in cured 0.5% agar with 0.5xMS after putting agar plugs into perforated plastic support f.) 0.5x MS is added to nutrient cup until contact is made between bottom of agar plug and MS solution g.) sterile water is added to height of inner nutrient cup level so nutrient cup does not have depletion of water h.) germination system with ~30 plants after 1 week from sowing seed.
(TIF)

S8 Fig Snapshots of platform assembly. a-b.) materials used in system assembly c.) 0.5xMS added to nutrient cup with LEGO^®^ brick support with perforated plastic sheet d.) paper pad is placed into cup and growth sheet is wicked across surface e.)rubber spacers are added onto each corner of paper surface
(TIF)

S9 Fig Final assembly steps. a-b.) plastic wrap is added to plastic seed support c.) plant from germination system is added by feeding root through central hole d.) sterile petroleum jelly is added to seal off agar plug thus preventing evaporation from plug. e-g.) plastic wrap is pressed against all side of nutrient cup like wrapping a gift and salt is added to control RH h.) a needle is added through both ports to allow access for sampling of cup and refilling of water i.) completed system with parafilm sealing around edges and over needle in port.
(TIF)

S10 Fig Shading of root by use of paper cover. (TIF)

S11 Fig Snapshots of root harvesting procedure. a-b.) the plastic wrap is removed from the system, the shoot is clipped from the root and the rubber spacers are removed. c.) the plastic sheet is used to invert the growth sheet with root d.) the growth paper is peeled away from the root unto instead the plastic sheet. e.) the plastic sheet is carefully removed to reveal the root geometry with contrasting background.
(TIF)

S12 Fig Growth chamber with paper systems. (TIF)

S13 Fig Spot testing for nutrient concentration experiments. a.)plastic sheet with testing sites b.)glass capillary pipette testing procedure
(TIF)

S1 Table Cost of system. (TIF)
