# Supplementary material for: Plant Growth Environments with Programmable Relative Humidity and Homogeneous Nutrient Availability
Source: PLoS One. 2016 Jun 15;11(6):e0155960. doi: 10.1371/journal.pone.0155960 (PMC4909320; doi:10.1371/journal.pone.0155960)
Supplement: S1 Methods — (DOCX) [file pone.0155960.s004.docx]

**Plant Growth Environments with Programmable Relative Humidity and Homogenous Nutrient Availability**

Kara R. Lind^1^, Nigel Lee^2^, Tom Sizmur^1,3,4^, Oskar Siemianowski^1^, Shawn Van Bruggen^1^, Baskar Ganapathysubramaniam^2^, Ludovico Cademartiri^1,4,5*^

^1^ Department of Materials Science & Engineering, Iowa State University of Science and Technology, Ames, IA, United States of America

^2^ Department of Mechanical Engineering, Iowa State University of Science and Technology, Ames, IA, United States of America

^3^ Department of Geography and Environmental Science, The University of Reading,

Reading, United Kingdom

^4^ Department of Chemical & Biological Engineering, Iowa State University of Science and Technology, Ames, IA, United States of America

^5^ Ames Laboratory, U.S. Department of Energy, Ames, IA, United States of America

^*^ Author to whom correspondence should be addressed: lcademar@iastate.edu

**Supporting Information**

**Assembly of the Experimental Apparatus (see S1 Movie)**

**Materials**

***Equipment***

Class II Biosafety Cabinet

Autoclave (Primus PSS5)

25 ml and 100 ml graduated cylinders

1 Liter glass bottles

25 ml glass vials

Analytical balance

Pipette 50-200 µl, pipette tips

Microwave

Oven capable of 80 °C

Digital Camera (Canon 50D, 100 Macro lens)

Paper cutter

Scissors

Clamp

Utility knife

Hygrometer (HMP42 Probe by Vaisala)

Drill with 3/16” 1/4” and 1” drill bits

Table saw or circular saw

***Apparatus and Consumables***

Whatman no. 1 filter paper (20cm X 20cm)

Choice of either:

White blotter paper (Grade 939 stultsscientific.com)

Whatman no. 3 filter paper (46cm X 57cm)

Lego^®^ bricks purchased from lego.com, Pick a Brick:

Round Brick – TR (Element ID: 3006840)

Sterilite^®^ flip top plastic container 7 5/8”x6 ½” x4 ½” (product code: 1803)

Polycarbonate sheeting 1/16” thick

Perforated polypropylene plastic sheeting (0.5 cm thick usplastics.com code: 42562)

Mainstays Food storage container 4”x4” or similar

Glad^®^ Press‘n Seal^®^ wrap

High temperature silicone rubber square bar 1/4” (product code 5508T42 mcmaster.com)

20/40 and 8mm septa

20cc syringe

20 gauge 6” needle

Sodium Chloride ( for 75% RH, use other salts for other RH)

Murashige & Skoog basal salt mixture with vitamins (product code: M519 from phytotechlab.com)

*Seeds: Brassica rapa* seeds (Wisconsin Fast Plants; Astroplants, dwf1)

Agar (product code: A111 from phytotechlab.com)

Petroleum jelly

Tweezers

Petri dish

Silicone sealant (transparent silicone plumbers caulk)

70% ethanol in spray bottle

Deionized water (DI water)

Bleach (Sodium hypochlorite 5.25% / di water (1:8 volume))

Aluminum foil

Autoclave indicator tape

Nitrile gloves

Ruler

Metal straight edge

50 ml centrifuge tube

Parafilm

Pipette tips used to make seedling plugs (200 µl universal Corning product code: 4862)

**Methods**

1. **Preparing paper components of apparatus**

White blotter paper (Grade 939) was purchased from Stults Scientific cut to 16 square inches. For each experimental apparatus, 7 sheets of paper are required in order to ensure that the thickness of the stacked sheets is larger than the distance between the holes in the perforated plastic sheet. If using Whatman #3, 12 sheets are required. Four Whatman #1 growth sheets are cut from a 20x 20 cm sheet using a paper cutter or scissors for conservation of materials. A single 100 cm^2^ Whatman #1 sheet is used for each experimental apparatus. (Note: when handling paper components wear gloves to prevent transfer of oils to paper)

1. **Preparing plastic components of apparatus**

While all materials are commercially available, amendments must be done initially to a few of the plastic components to make them suitable for the experimental apparatus. After these initial changes, these components are reusable in future experiments. To give plants more headspace, the nutrient cup walls are cut from the Mainstays Food storage container using a scissors so that the cup walls are 3 cm tall.


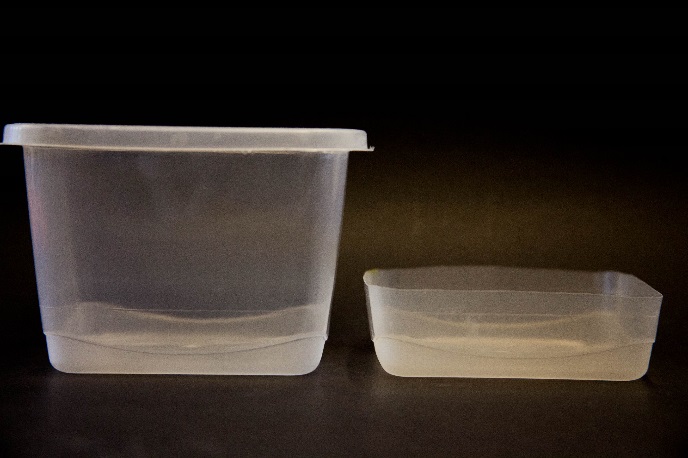


**S1 Fig. Nutrient Cup before and after cutting walls of container**

Using a table saw or circular saw, the perforated plastic sheeting is cut to the desired sizes (see Fig 2S). Appropriate training, assessment of risk and control measures should be put in place when using these tools. For our system, these perforated polypropylene plastic sheets were cut to be 16 square inches. In order to fit into the nutrient cup container, a paper cutter was used to clip the corners since the nutrient cup has slightly rounded corners. Additionally, a hand drill with a 3/16” drill bit is used to enlarge a hole near each corner to ensure a snug fit of the Lego^®^ bricks.


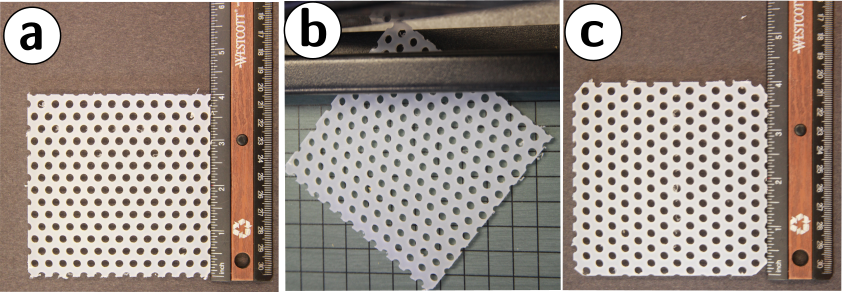


**S2 Fig. Fabricating perforated plastic support** a.) cut perforated polypropylene sheeting b.) rounding corners using paper cutter c.) finished perforated sheeting for platform

To cut the thin polycarbonate plastic sheeting used for the seed support, a utility knife is used. While a table saw could be used this method is recommended as it results in less wasted material. Using a large ruler, measure and trace out the size required for the experimental apparatus. In our case, we cut the plastic sheeting to be 4.5 square inches so that the plastic would provide a viewing window of the entire paper sheet. Attach the large plastic sheet to a table using a clamp. Using a metal straight edge, score on the traced lines several times. Once the plastic has been scored many times, the plastic sheeting can be snapped into two pieces easily. After cutting the plastic sheet to the correct size, drill a 3/16” hole in the center of the sheet which will be used for the agar plug. An additional 1/4” hole is drilled as close to the one edge as possible and 1” from the other edge as shown in Fig 3Sg. A 8 mm septum is then placed into this hole. This will act as port for the system.


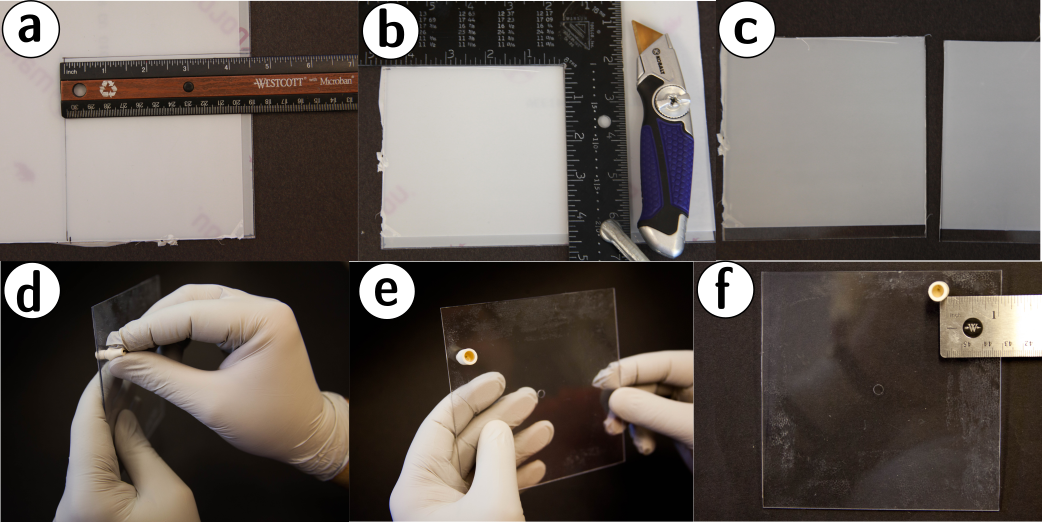


**S3 Fig. Polycarbonate plastic sheeting amendments** a.) Traced out size for platform b.) Clamp sheet to table with metal straight edge and score plastic with utility knife c.) Snap plastic into two pieces d-f.) Septum attached to plastic sheet g.) Finished polycarbonate plastic sheeting for platform

The external container (Sterilite^®^ flip top plastic container) has a single 1” drill hole for a 20/40 septum. This should be placed 1” and 1/2” from the viewing window of the external container as shown in Fig S4. Use the silicone caulk to seal the septum to the external container. Allow silicone caulk to cure overnight before autoclaving


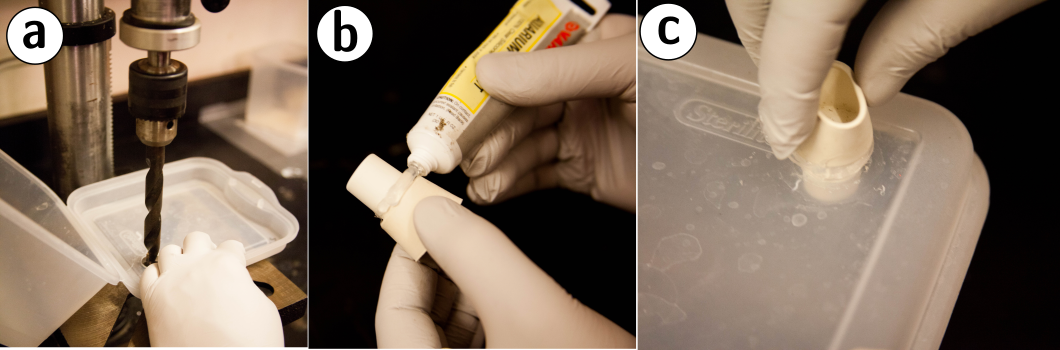


**S4 Fig. Modifications to external container** a.) 1” hole drilled into external container lid b.) Placement of port

To prepare the agar plug. A 200 µl pipette tip is cut using a scissors. This plug size works for a number of seed sizes. Spacers are made by cutting silicone rubber square bar to ¼ inch lengths using a utility knife. Spacers will be placed on the paper surface to provide a gap between the polycarbonate plastic sheet and paper surface.


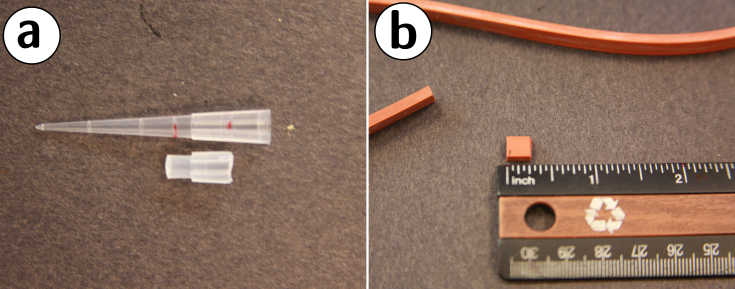


**S5 Fig. Preparation of plug and spacers** a.)Before cutting and after cutting pipette tip for agar plug b.) Silicone spacers

1. **Preparing system for sterilization**

Inside each external container, place a nutrient cup. Take 8 round LEGO^®^ bricks (Element ID:3006840) and create 4 columns by attaching two LEGO^®^ bricks together. Attach each column to the perforated plastic sheet at the drilled corners to create the perforated LEGO^®^ support for the experiment. On top of this place 7 sheets of 4 X 4 inch white blotter paper and 1 sheet of 10 square centimeter Ehatman #1 paper. Use 12 sheets of Whatman #3 filter paper if not using blotter paper. Finally, include the 4 silicone spacers and polycarbonate plastic sheet seed support with agar plug. Close the external container lid and add a small piece of autoclave indicator tape. Autoclave the system for 3.5 minutes at 132 °C under the vacuum cycle.
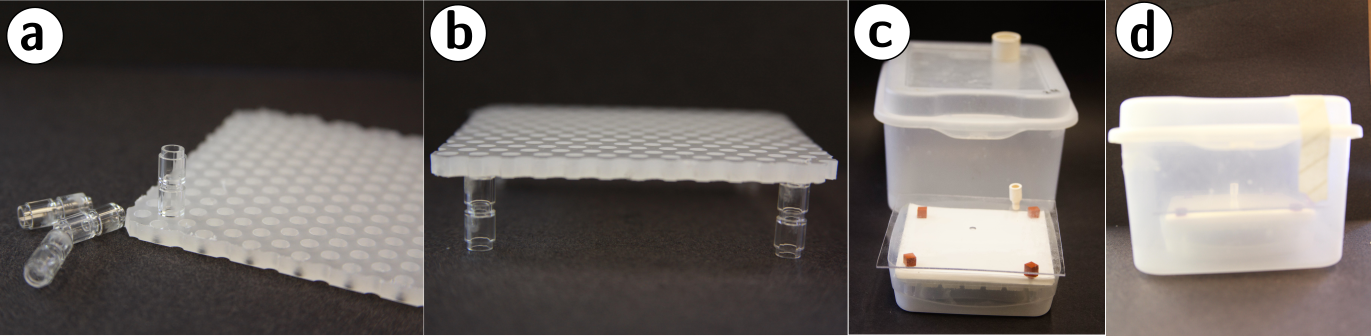


**S6 Fig. Preparing system for sterilization** a.) Assembly of perforated LEGO^®^ support b.) Finished LEGO^®^ support c.) Preparing system for autoclaving d.) System ready for sterilization

1. **Nutrient solution and Nutrient gel**

2.215 g of Murashige & Skoog (MS) Basal Medium w/Vitamins is added to 1 Liter of DI water inside a glass bottle (2.215 g/L=x0.5MS). Without fully tightening the bottle, add a small piece of autoclave indicator tape and autoclave for 15 minutes at 121 °C using the Primus cycle 6 liquid cycle.

Using the analytical balance, measure 50 mg of Agar and 22.15 mg MS. Dissolve the MS and agar in 10 ml (0.5%) of DIwater in a 25 ml glass vials (50mg/ml = 0.5%). The cap is closed without tightening with autoclave indicator tape. The Nutrient gel is then autoclaved similarly to the nutrient solution.

1. **Seed pretreatment and sterilization**

Surface sterilization is required for *Brassica rapa* seeds (Wisconsin Fast Plants; Astroplants, dwf1) When ready to plant seeds, place *Brassica rapa* seeds in a petri dish and cover with 70% ethanol. Allow ethanol to evaporate in biosafety cabinet. Prepare 45 ml of dilute bleach solution by combining 5 ml of bleach and 40 ml of DI water into a 50 ml centrifuge tube. Place seeds in petri dish and pour 15 ml of bleach to cover seeds. Incubate seeds for 5 minutes in biological safety cabinet before washing with sterile water. Wash with sterile water 3 times before planting seeds into agar plugs.

1. **Preparing agar plugs for experiment**

Agar plugs containing seedlings can be prepared prior to each experiment and introduced into our growth environments to begin an experiment with 100% germination. Plants can also be germinated directly in the system only if the relative humidity is high (i.e. 95%) otherwise the agar will become desiccated before the plant root touches the paper surface.

Place the glass vial with slightly loose lid containing sterile agar in the microwave for ~10 seconds to bring vial contents into solution and shake. Introduce the vial into the biosafety cabinet by spraying with 70% ethanol solution. Allow agar to slightly cool. Then, using a micropipette, inject 300 µl of agar into each plug, using parafilm to seal the bottom end. Once the agar has set, add desired seed. (Note: plugs are cut following the procedure outlines in Fig S5a and autoclaved before adding agar and seed)

When the intent is to study more than one system, a batch assembly scheme can be used that germinates seeds outside the paper platform inside the germination system (S2 Movie). The germination system is composed using pieces from the whole system. This method produces 30 *Brassica rapa* plants that are germinated under the same initial conditions (e.g. light intensity and nutrient concentration). Once the seeds have germinated and grown to the desired level, individual plugs with individual plants are transferred to individual paper systems.


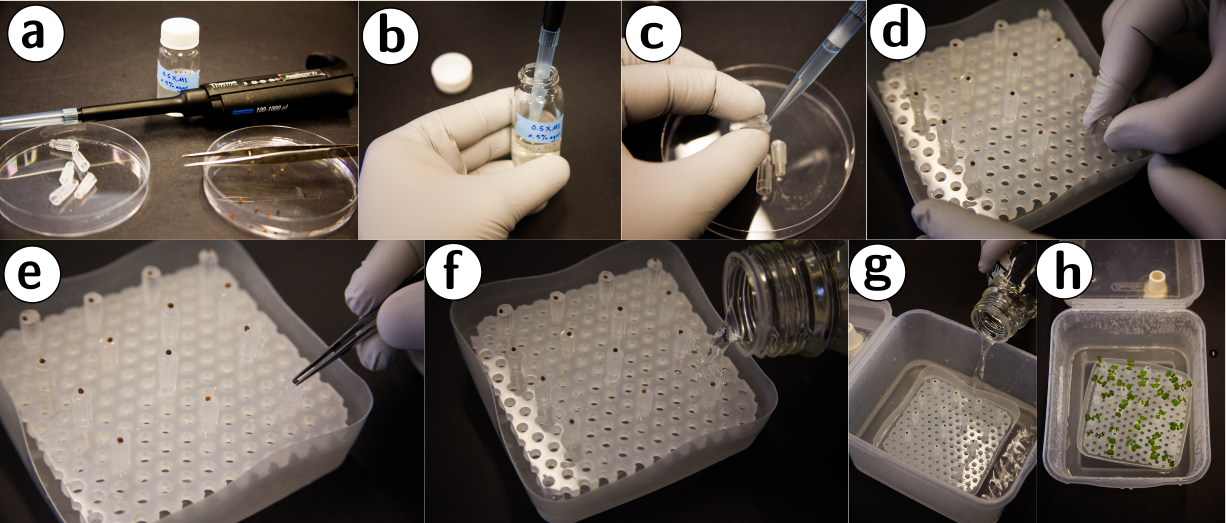


**S7 Fig. Agar plug assembly for multiple systems intended to study germination and growth** a-e.) Sterilized brassica seeds are placed in cured 0.5% agar with 0.5xMS after putting agar plugs into perforated plastic support f.) 0.5x MS is added to nutrient cup until contact is made between bottom of agar plug and MS solution g.) sterile water is added to height of inner nutrient cup level so nutrient cup does not have depletion of water h.) germination system with ~30 plants after 1 week from sowing seed.

1. **Introducing materials into the biosafety cabinet**

Wearing nitrile gloves, ensure the biosafety cabinet is clean by spraying with 70% ethanol. Everything that has been autoclaved (the experimental apparatus, measuring cylinders wrapped with aluminum foil, the plastic bottles containing the salt, and glass bottles containing the nutrient solution) is sprayed with 70% ethanol before placing the biosafety cabinet. The required number of seeds is placed on a petri dish after being sterilized according to procedure previously mentioned if using seeds rather than germinated plants.

1. **Assembling experimental platform (see Movie S1)**

Once materials have been introduced into the biosafety cabinet, remove autoclave tape and aluminum foil and dispose. Take the nutrient cup and other components out of the external container. Place the perforated Lego^®^ support inside the nutrient cup and fill with 220ml 0.5xMS nutrient solution. Place the white blotter paper back on the Lego^®^ support and pour an additional 50ml of nutrient solution on top. Wick the Whatman #1 paper growth sheet by beginning at one end of the white blotter pad. Place silicone bars on the corners of the top paper sheet.


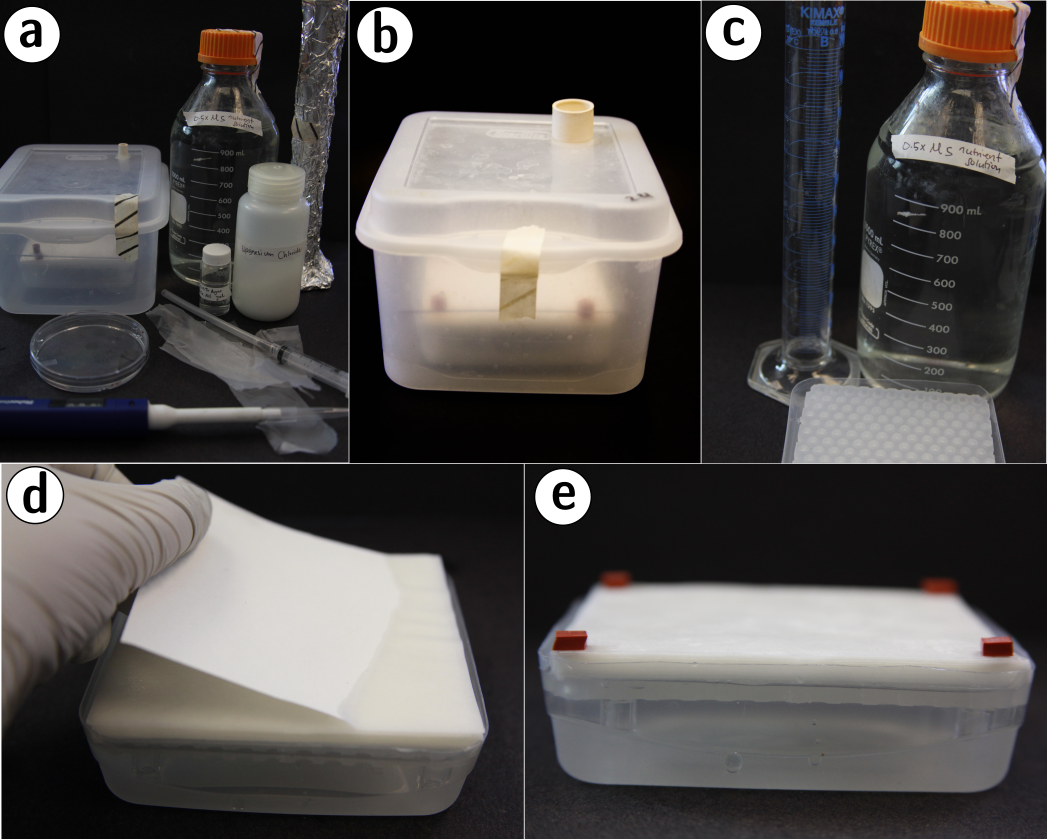


**S8 Fig. Snapshots of platform assembly** a-b.) materials used in system assembly c.) 0.5xMS added to nutrient cup with LEGO^®^ brick support with perforated plastic sheet d.) paper pad is placed into cup and growth sheet is wicked across surface e.)rubber spacers are added onto each corner of paper surface

Four small strips of Glad^®^ Press‘n Seal^®^ wrap (~5 inch by 3 inch) are added to each edge of the polycarbonate seed support. Using your fingers, press along the strips to attach them to the polycarbonate seed support on all four edges. Once the strips are attached, place seed support so it is resting on top of the silicone bars. Make any necessary adjustments such as ensuring that the port is accessible to the nutrient solution below. Seal the nutrient cup with the plastic wrap strips like wrapping a present.

Add 100 ml of saturated salt solution to the external container for relative humidity control and 50 grams of crystalline salt for salts that are above the relative humidity value of the room’s air. For salts that are below the relative humidity of the air use only 100 grams of crystalline salt and no saturated solution. Place the nutrient cup inside the external container. Close lid of external container and insert a single syringe needle through both the external and internal ports. Apply Parafilm around the lid and over the needle and port to seal the box before removing it from the biosafety cabinet and placing in the growth chamber
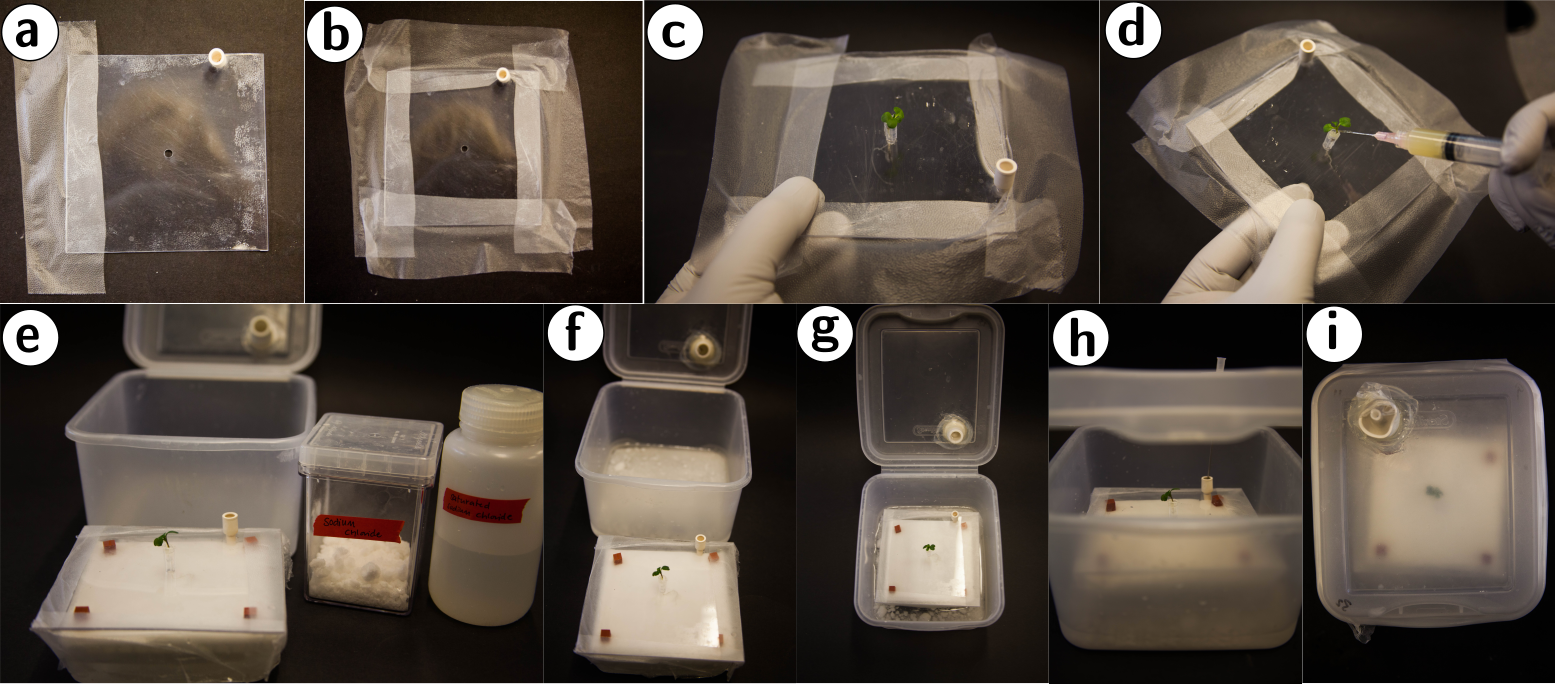
 **S9 Fig. Final assembly steps** a-b.) plastic wrap is added to plastic seed support c.) plant from germination system is added by feeding root through central hole d.) sterile petroleum jelly is added to seal off agar plug thus preventing evaporation from plug. e-g.) plastic wrap is pressed against all side of nutrient cup like wrapping a gift and salt is added to control RH h.) a needle is added through both ports to allow access for sampling of cup and refilling of water i.) completed system with parafilm sealing around edges and over needle in port.

1. **Shading of root capability**

When the researcher would like shading of the root, 2 pieces of Whatman #3 paper cut and a piece of aluminum foil are placed are used. The aluminum foil and Whatman #3 paper are cut to the size of the plastic sheet that supports the seedling. The paper sandwiches the aluminum foil and small holes are punched to match the plant plug position and port position. Note: the plastic wrap was removed prior to imaging in Fig 13S below.


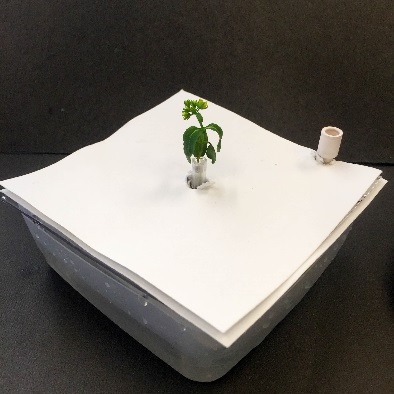


**S10 Fig. Shading of root by use of paper cover**

1. **Harvesting Root from system for phenotype imaging**

The root can be easily imaged using by using the plastic sheet. First, the shoot is cut from the root just under the plug using scissors. The root will become fully visible on the paper surface at this point. The rubber spacers are removed from the surface and the growth sheet is inverted onto the plastic sheet. The root can then be easily transferred to the plastic sheet by carefully pealing the growth sheet away. The plastic sheet with the root on top can then be laid over black paper for greater contrast and imaged directly through the plastic sheet. Otherwise, the plastic sheet can be removed to leave only the root on the black paper. A ruler is then included in the image for later scaling necessary for root phenotyping.
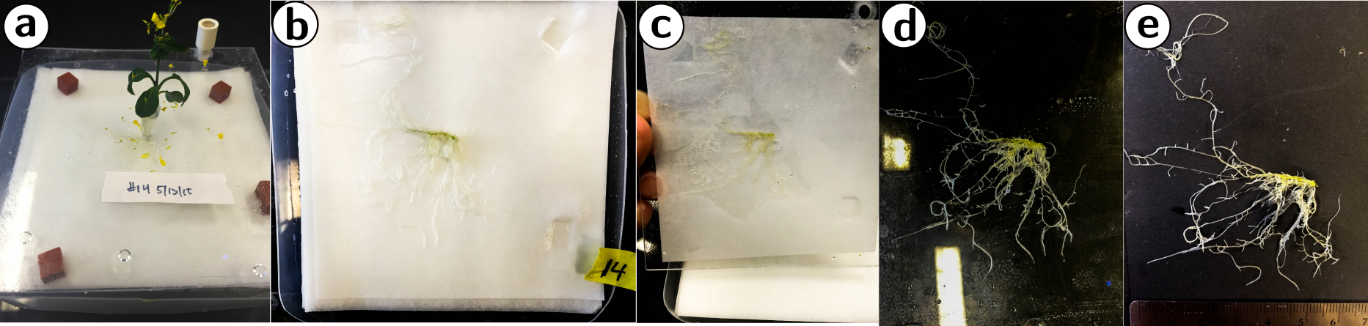


**S11 Fig. Snapshots of root harvesting procedure** a-b.) the plastic wrap is removed from the system, the shoot is clipped from the root and the rubber spacers are removed. c.) the plastic sheet is used to invert the growth sheet with root d.) the growth paper is peeled away from the root unto instead the plastic sheet. e.) the plastic sheet is carefully removed to reveal the root geometry with contrasting background.

1. **Growth chamber specifications**

The growth chambers were custom built for the purpose of providing uniform illumination. Industrial shelving was constructed. Each shelf is 3 ft. by 8 ft. with 10 LED panels evenly spaced over shelf. The LED is hanging from the top of the chamber at a height to provide a ~140 PAR ± 10 PAR. The temperature of the room is controlled by air conditioning to provide an internal room temperature of 20-23°C.


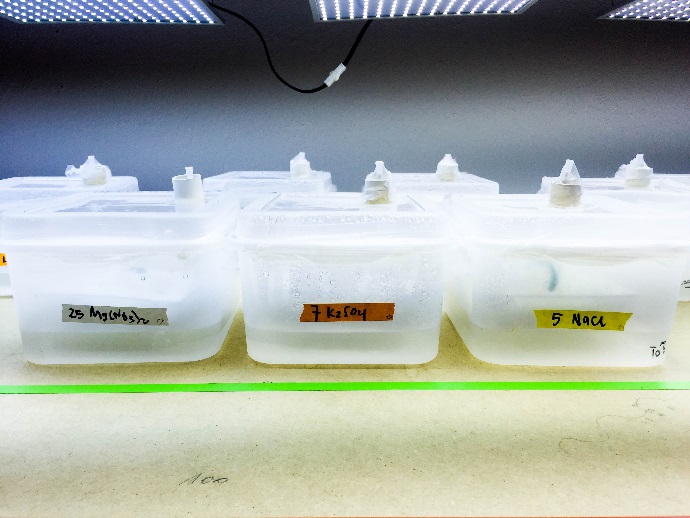


**S12 Fig. Growth chamber with paper systems**

1. **Cost estimates**

| Item | Cost (4 platforms) | Cost per platform | Reusable | Disposable |
| --- | --- | --- | --- | --- |
| Whatman no. 1 filter paper | $0.46 | $0.12 |  |  |
| blotter paper used for pad | $1.74 | $0.43 |  |  |
| LEGO® bricks: round tall | $3.60 | $0.90 | $0.90 |  |
| septa for water refilling | $6.20 | $1.05 | $1.05 |  |
| Sterlite External Container | $4.00 | $1.00 | $1.00 |  |
| polycabonate cover | $1.72 | $0.43 | $0.43 |  |
| Internal Nutrient Container | $1.47 | $0.37 | $0.37 |  |
| Silicone rubber spacers | $0.03 | $0.01 | $0.01 |  |
| Preforated Plastic Sheet | $2.10 | $0.53 | $0.53 |  |
| Sodium Chloride | $4.00 | $1.00 | $1.00 |  |
| parafilm | $0.84 | $0.21 |  |  |
| silcone chaulk | $0.08 | $0.02 | $0.02 |  |
| plastic film (Gladware) | $0.38 | $0.09 |  |  |
| Murashige & Skoog (MS) | $0.20 | $0.05 |  |  |
| Agar | $0.03 | $0.01 |  |  |
| petroleum jelly | $0.01 | $0.01 |  |  |
| Ethanol | $0.21 | $0.05 |  |  |
| pipette tip used as agar plug | $0.06 | $0.02 | $0.02 |  |
| syringe 20 CC | $1.04 | $0.26 | $0.26 |  |
| needle 20 gauge 6" | $4.52 | $1.13 | $1.13 |  |
| Brassica rapa seeds | $0.59 | $0.15 |  |  |
|  | Totals without seed | $7.24 | $6.70 | $0.54 |
|  | Totals with seed | $7.39 | $6.70 | $0.69 |

**S1 Table: Cost of system**

**Experimental Design**

**Nutrient Concentration Experiments**

1. We assembled 5 paper platforms until the point of adding the growth sheet (Whatman #1) using a blotter paper pad and 5 more systems using Whatman #3 paper pad ( Note: in this study 2 blotter systems did not survive transplant resulting in n=3 for blotter pads and n=5 for Whatman #3 pads)
2. A growth sheet (i.e. Whatman #1) was placed on each system.
3. Systems with all components necessary for assembly were autoclaved. Note: the plastic seed support was amended by drilling additional ¼” holes in the pattern in Fig 16S. Holes were covered by using additional sheets of plastic wrap across top surface to prevent evaporation from root environment.
4. In biosafety cabinet, the system was assembled and a 7 day old plants were transplanted from the germination system into the paper system. 1.5 ml samples of nutrient cup solution were taken immediately after assembly using the syringe port and stored in sterile Eppendorf tubes until later testing.
5. Assembled systems were placed into growth chamber under continuous light (~140 PAR) for ~2 weeks.
6. Samples were taken from nutrient cups on 24 hours, 7 days, 11 days, and 13 days after assembling system with plants. Throughout the study DI water is added to systems to maintain the nutrient cup concentration level thus preventing accumulation of nutrients on paper surfaces.
7. Samples of paper surface were taken on the same days using a glass capillary pipette on spots 1-6 shown in Fig 16S. ~50 microliters of solution was taken from paper surface and diluted with sterile water and stored for later testing from each spot of each system.


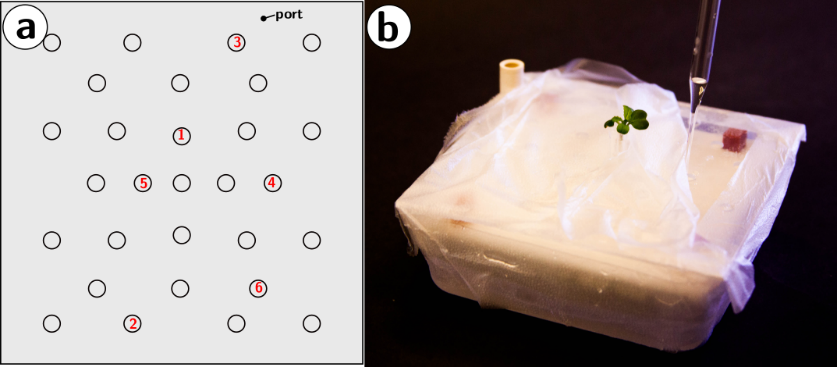


**S13 Fig. Spot testing for nutrient concentration experiments** a.)plastic sheet with testing sites b.)glass capillary pipette testing procedure

1. Samples were tested using either ICP-AES (K^+^ and P^+^ concentrations ) or a Lachat flow analyzer ( NH4^+^ and NO3^-^ concentrations)
2. The concentration of the nutrient cup with time was compared to that of the paper surface concentrations for all spots.
3. The concentration gradient was determined for ions estimating the accumulation rate of that ion at steady state if the system is not refilled.
4. The variance from spot to spot was determined for the ions (the root mean square) as well as the difference in concentration of spots near the root compared to away from the root.

**Determination of Concentration Gradient**

The concentration gradinet in our system at steady-state can be determined using the 1-D solution to Fick’s 1^st^ law of diffusion

$$J=-D\frac{\Delta[C]}{\Delta z}$$

Where

- J is the diffusive flux expressed in units of $\frac{mol}{{cm}^{2}\cdot s}$
- D is the diffusion coefficent in units of $\frac{{cm}^{2}}{s}$
- Δ[C] is the concentration gradient in units of $\frac{mol}{{cm}^{3}}$
- Δz is the distance over which the flux occurs in units of $cm$

The flux is first determined by considering the water loss from the nutrient cup. This flux is the combined result of flow of water from evaporation and transpiration (J_evap,H2O_ + J_transp,H2O_). In our case, the average result was J_evap,H2O_ + J_transp,H2O_ = 4+1 ml/ 100 cm ^2^ paper ·day for each system. These fluxes together we will define here J_H2O_ which can then be equated to J_evap,nutrients_ . J_evap,nutrients_ = J_H2O_*[C]*FW/1000, where [C] is the molarity of the nutrient in mol/l, FW is the formula weight in g/mol). J_evap,nutrients_ is directly matched by the downward flow of nutrients which we call J_diff_.

From J_evap,nutrients_  or J_diff_ the concentration gradient is determined with the known diffusivity of the ion of interest[1] and the initial concentration of the ion in the nutrient cup. An estimate of each ions accumulation at steady state can then be determined.

Sample calculation for Phosphorus at steady state

$$J_{H_{2}O=Jevap,H2O + Jtransp,H2O=}=\frac{\frac{1+4 ml H_{2}O}{day}}{}100 {cm}^{2}paper=0.05\frac{{ml H}_{2}O}{{cm}^{2}\cdot day}$$

$$=5E^{-5}\frac{{L H}_{2}O}{{cm}^{2}\cdot day}$$

$$mass flow of P=\frac{20.45 mg P}{L}*\frac{1 g P}{1000 mg P}*\frac{1 mol P}{30.97 g P}=.00066\frac{mol P}{L}$$

$$J_{evap,P}=5E^{-5}\frac{{L H}_{2}O}{{cm}^{2}\cdot day}*0.00066\frac{mol P}{L}*\frac{30.97 g P}{1 mol P}=1.02E^{-6}mg P/{cm}^{2}\cdot day$$

$$J_{evap,P}=-D\frac{\Delta\left[ C \right]}{\Delta z}$$

$$J_{evap,P}=1.02E^{-6}mg\frac{P}{{cm}^{2}}\cdot day=-\frac{0.89E^{-5}{cm}^{2}}{s}*\frac{\Delta\left[ C \right]}{0.2 cm}$$

Solve for Δ[C]

$$\Delta\left[ C \right]=.00026 \frac{mg P}{{cm}^{3}}=0.27\frac{mg}{L}$$

Using the initial P concentration of nutrient cup and the calculated Δ[C] determine the estimated accumulation of P at steady state

$$\% accumulation P={\left( 20.45\frac{mg}{L}+0.27\frac{mg}{L} \right)-20.45\frac{mg}{L}}/{20.45\frac{mg}{L}*100\%=1.3\%}$$

**Variance calculations**

The variance of from spot to spot on the paper growth sheet was determined for all ions. This calculation is based on the the the average variance of all ions. The variance of ions was also determined based on the proximity to the root, i.e. away from the root or near the root. Away from the root was only considered if it was at least 5 mm away from any root in any direction. 6 spots where sampled on each pad of each system. The Root mean square (RMS) was determined for all systems used in the plant study to give indication of the error of concentration from spot to spot.

The relative variance of nutrients on the growth sheet considers each spot with the corresponding pad system using the following equation:

$relative variance of each spot =\frac{\left[ ion concentraiton at spot \right]-[average ion concentration over that pad]}{[average ion concentration over that pad]}*100\%$

Example Calculation for Whatman system 3

$$average {NH}_{4}^{+}concentration on pad=126.58 ppm$$

$${NH}_{4}^{+}concentration at spot 1=126.25 ppm$$

$$relative variance of {NH}_{4}^{+} at spot 1 =\frac{\left[ 126.25ppm \right]-[126.58 ppm]}{[126.58 ppm]}*100\%=\boldsymbol{-0.2607\%}$$

An average variance was determined for the proximity away from the root over all systems by averaging all relative variances of spots of all systems that were at least 5 mm away from any root. The average relative variance was also determined for all spots near the root for all systems. The average relative variance near and away from the root is tabluated below.

| Spot proximity to root | Average Relative Variance | Standard Error |
| --- | --- | --- |
| **Away from root** | 0.2938 % | 0.0109 |
| **Near root** | -0.2908 % | 0.0192 |

The root mean square (RMS) was determined for all spots for all ions across all pad systems.

The square mean of each ion’s relative variance was determined by the following equations

$${{Mean}^{2}}_{{NH}_{4}^{+}}={(({relative variance of {NH}_{4}^{+}}_{spot 1, system 1})}^{2}+{{(relative variance of {NH}_{4}^{+}}_{spot 2, system1})}^{2}+\ldots+{{(relative variance of {NH}_{4}^{+}}_{spot n, system n})}^{2}$$

$${{Mean}^{2}}_{{NO}_{3}^{-}}={(({relative variance of {NO}_{3}^{-}}_{spot 1, system 1})}^{2}+{{(relative variance of {NO}_{3}^{-}}_{spot 2, system1})}^{2}+\ldots+{{(relative variance of {NO}_{3}^{-}}_{spot n, system n})}^{2}$$

$${{Mean}^{2}}_{P^{+}}={(({relative variance of P^{+}}_{spot 1, system 1})}^{2}+{{(relative variance of P^{+}}_{spot 2, system1})}^{2}+\ldots+{{(relative variance of P^{+}}_{spot n, system n})}^{2}$$

$${{Mean}^{2}}_{K^{+}}={(({relative variance of K^{+}}_{spot 1, system 1})}^{2}+{{(relative variance of K^{+}}_{spot 2, system1})}^{2}+\ldots+{{(relative variance of K^{+}}_{spot n, system n})}^{2}$$

The RMS was determined by

$$RMS=\sqrt{\frac{1}{n}{{(Mean}^{2}}_{{NH}_{4}^{+}}+{{Mean}^{2}}_{{NO}_{3}^{-}}+{{Mean}^{2}}_{P^{+}}+{{Mean}^{2}}_{K^{+}}})$$

Where n is the sum of all relative variances for all ions in the study

| **Ion** | **n** | **Mean Square** | **RMS** |
| --- | --- | --- | --- |
| NH_4_^+^ | 166 | 161.875 % |  |
| NO_3_^-^ | 166 | 224.995 % |  |
| P^+^ | 164 | 246.821 % |  |
| K^+^ | 160 | 284.949 % |  |
| ***All ions*** | ***656*** | ***91863.926 %*** | ***11.834 %*** |

**Relative Humidity Experiments**

**Determining the range of Relative Humidity capable in our system**

1. We assembled paper systems using various supersaturated salt solutions and crystalline salts without plants.
2. Saturated salt solutions where prepared by dissolving the amount of salt listed below into 100 ml of DI water to produce a wide range of relative humidity capable in our system. When the room RH was higher than the desired RH, crystalline salt was added in excess to reduce RH and prevent immediate consumption of salts. For our experiments, we used crystalline salt only for systems where we wanted the RH below 50%.

| **Salt** | **mass (g) per 100 ml Water** | **Literature RH (%)** |
| --- | --- | --- |
| Lithium Chloride | 115 | 11.3 |
| Potassium Acetate | 268.6 | 23 |
| Magnesium Chloride | 220 | 33 |
| Potassium Carbonate | 224 | 44 |
| Magnesium Nitrate | 125 | 55 |
| Sodium Bromide | 94.23 | 57.9 |
| Sodium Nitrate | 91.2 | 66 |
| Sodium chloride | 72 | 75.7 |
| Potassium chloride | 80 | 85 |
| Potassium nitrate | 164.4 | 93 |
| Potassium sulfate | 50 | 97 |

1. We then waited 24 hours to allow systems to reach equilibrium relative humidity.
2. We pierced the large exterior septa on system and inserted hygrometer probe so that tip of the probe is 2 cm from the top of the plastic sheet.
3. We then waited till hygrometer has stabilized and record temperature and relative humidity.
4. The result of this experiment is found in Fig 2B of the manuscript.

**Determining the programmability of relative humidity with incorporation of plants**

Measurements of plants with sodium chloride (75% RH) for relative humidity control were tested with the incorporation of the plants in these systems over a 2 week period. The results of this were discussed in the manuscript and in Fig 2c. The difference in relative humidity is likely the result of imperfect sealing of the system from the lab atmosphere.

**Root phenotyping as function of programmed relative humidity**

1. The root phenotyping experiments were conducted by using 3 salts to produce 3 distinct relative humidity values. We have found that brassica plants grown below 55% where unable to survive due to extreme desiccation and transpiration of the plant (we tried growing plants at RH=33% and 44% without success).
2. Plants were transplanted from the same germination system into the paper systems and left for 24 hours under 100% to reduce transplant shock from the germination system. These systems were placed under continuous light after 2 hours of assembly.
3. After 24 hours, systems were programmed to the desired relative humidity by the incorporation of saturated salt solutions. For systems of 55%, only crystalline magnesium nitrate (50g) was added to the box. For 75% and 95%, saturated solutions and 50g of crystalline salt were added to the systems. Sodium chloride and potassium nitrate were used respectively for 75% and 95%.
4. Relative humidity and temperatures of these systems were taken over a 2 week period by piercing the outer septa with the hygrometer.
5. Note: if the system is checked the relative humidity is not close to the set point (likely due to plastic wrap becoming unsealed from the nutrient cup) , additional crystalline salt can be added to regain the set point.
6. The roots of these systems were harvesting using our procedure and imaged using a digital camera (Canon DSLR). The root phenotyping was completed by use high-throughput image analysis tool ARIA (Automatic Root Image Analysis)[2].
7. From ARIA, phenotyping dimensions of total surface area, width, depth, width/depth ratio, and convex area were considered.
8. In addition to ARIA, ImageJ was used to compute the void sizes across all plants. Specifically:
   1. A scale was set for the image with the help of a ruler within the photograph.
   2. The image was cropped to include only the root system
   3. The contrast and brightness and gamma setting were adjusted by hand to help thresholding.
   4. The images were converted to 8-bit
   5. The images were thresholded by hand by the “over-under” setting, yielding a figure were the background’s color value was 255 and the root’s color value was 0.
   6. The “analyze particles” was then used to yield the size of all “voids”.
   7. The areas were then binned with bins uniformly spaced in the log scale. Data from areas corresponding to linear dimensions smaller than 300 micrometers were discarded.
   8. The binned data were normalized and plotted for the 55%, 75%, and 95%RH plants.
9. The dry biomass of roots and shoots of systems was determined by weighing individual samples after drying overnight in 80°C oven.

**References**

1. Haynes WM. CRC handbook of chemistry and physics: CRC press; 2014.

2. Pace J, Lee N, Naik HS, Ganapathysubramanian B, Luebberstedt T. Analysis of Maize (Zea mays L.) Seedling Roots with the High-Throughput Image Analysis Tool ARIA (Automatic Root Image Analysis). Plos One. 2014;9(9). doi: 10.1371/journal.pone.0108255. PubMed PMID: WOS:000342492700103.
