# Supplementary figures and images for: Plant Growth Environments with Programmable Relative Humidity and Homogeneous Nutrient Availability
Source: PLoS One. 2016 Jun 15;11(6):e0155960. doi: 10.1371/journal.pone.0155960 (PMC4909320; doi:10.1371/journal.pone.0155960)

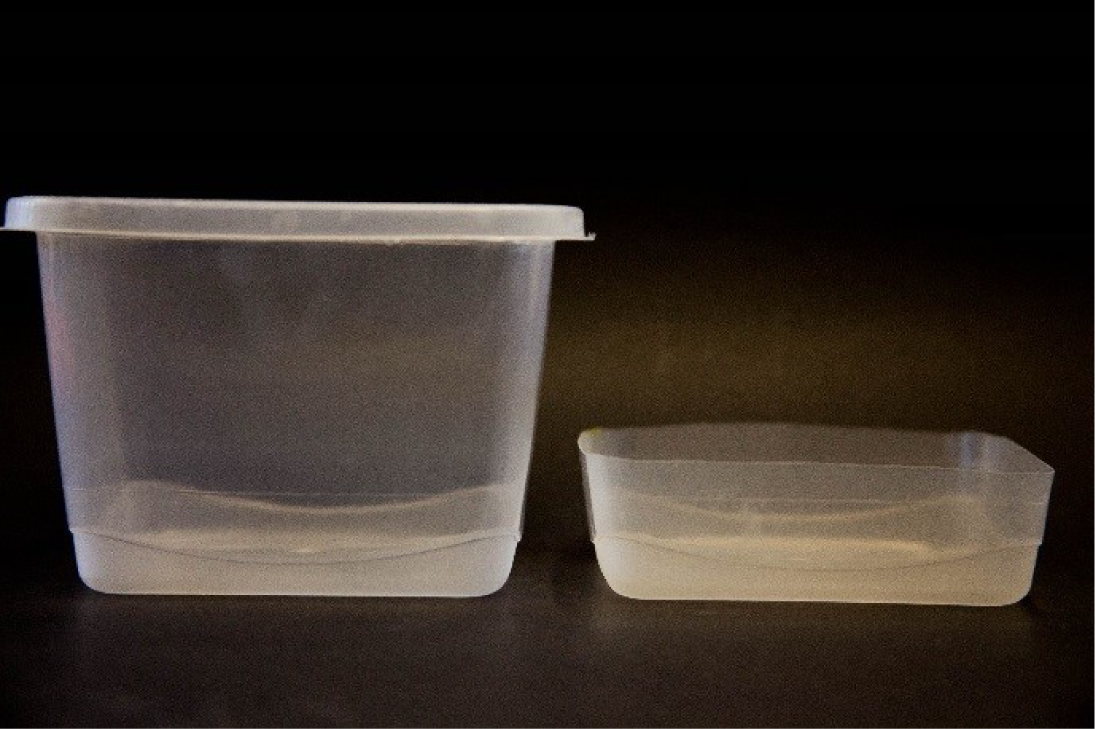

Supplement: S1 Fig — (TIF) [file pone.0155960.s005.tif]

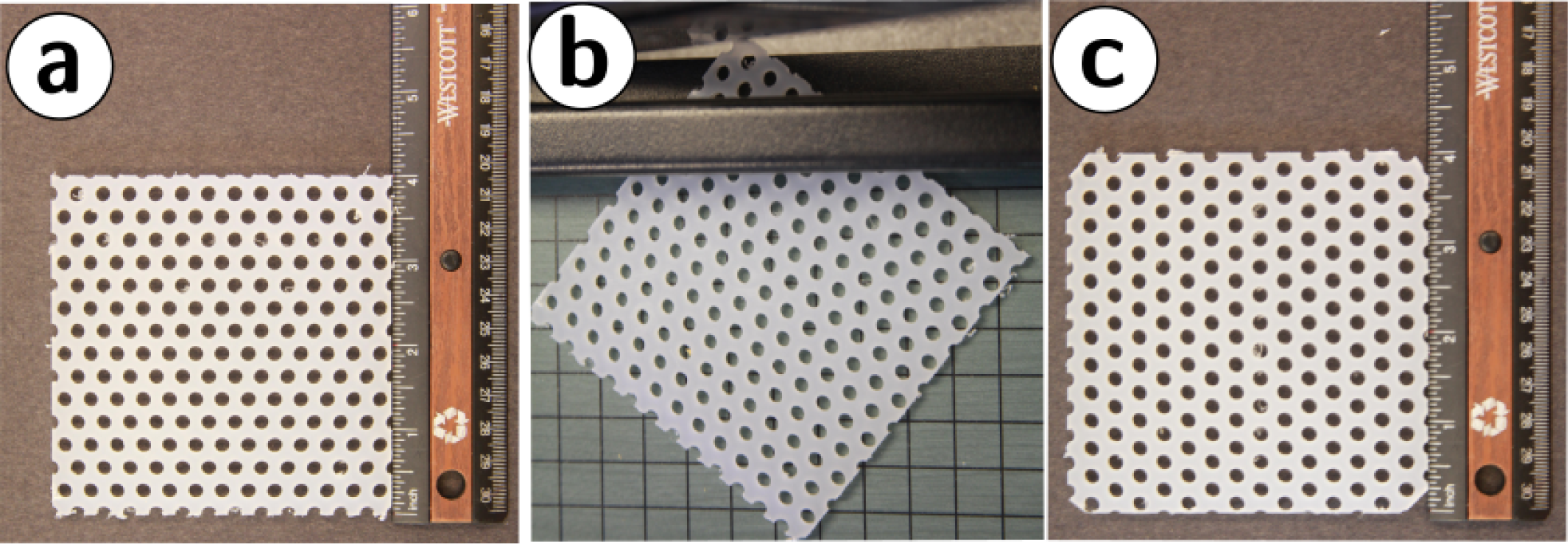

Supplement: S2 Fig — a.) cut perforated polypropylene sheeting b.) rounding corners using paper cutter c.) finished perforated sheeting for platform (TIF) [file pone.0155960.s006.tif]

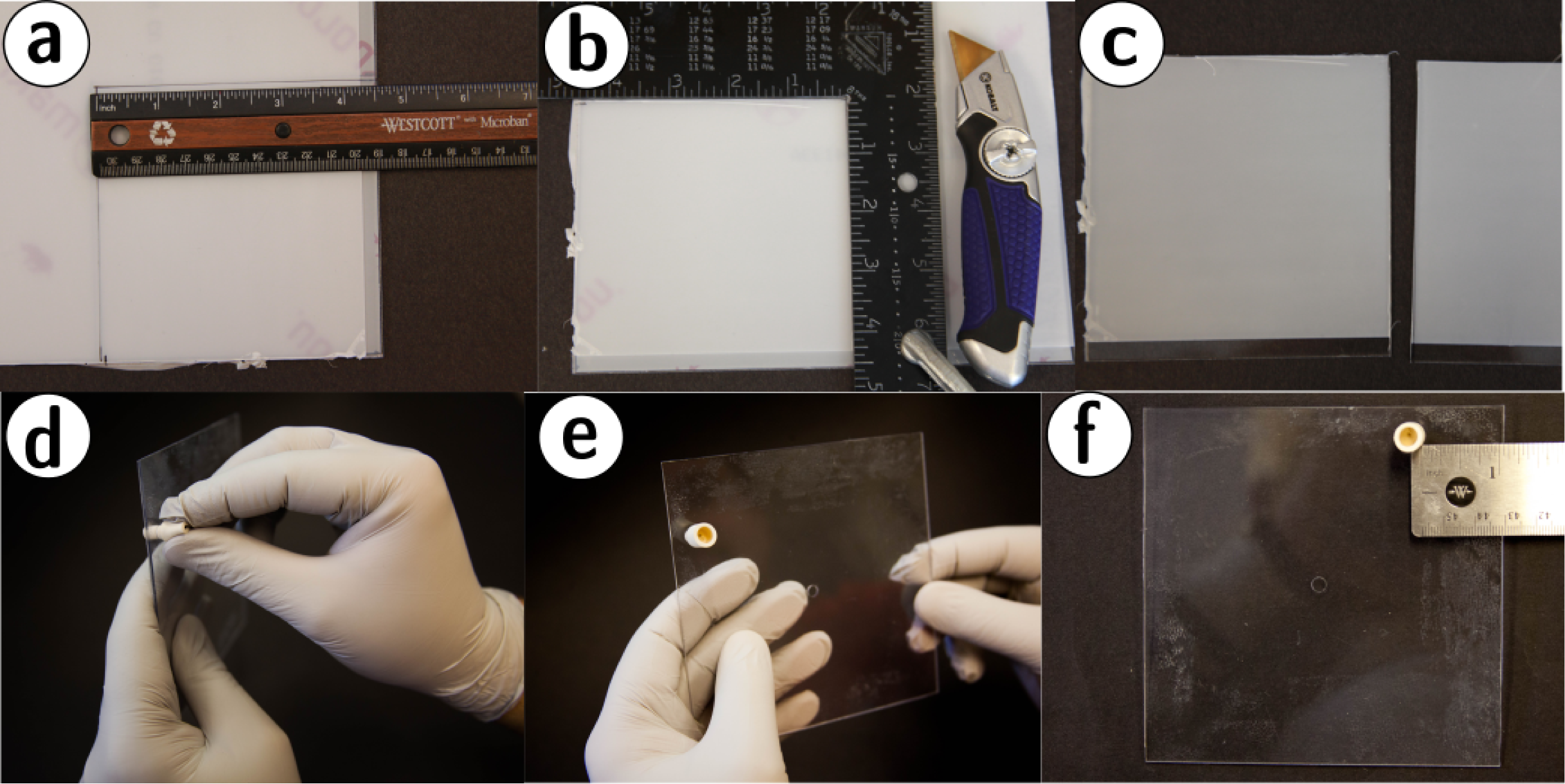

Supplement: S3 Fig — a.) Traced out size for platform b.) Clamp sheet to table with metal straight edge and score plastic with utility knife c.) Snap plastic into two pieces d-f.) Septum attached to plastic sheet g.) Finished polycarbonate plastic sheeting for platform (TIF) [file pone.0155960.s007.tif]

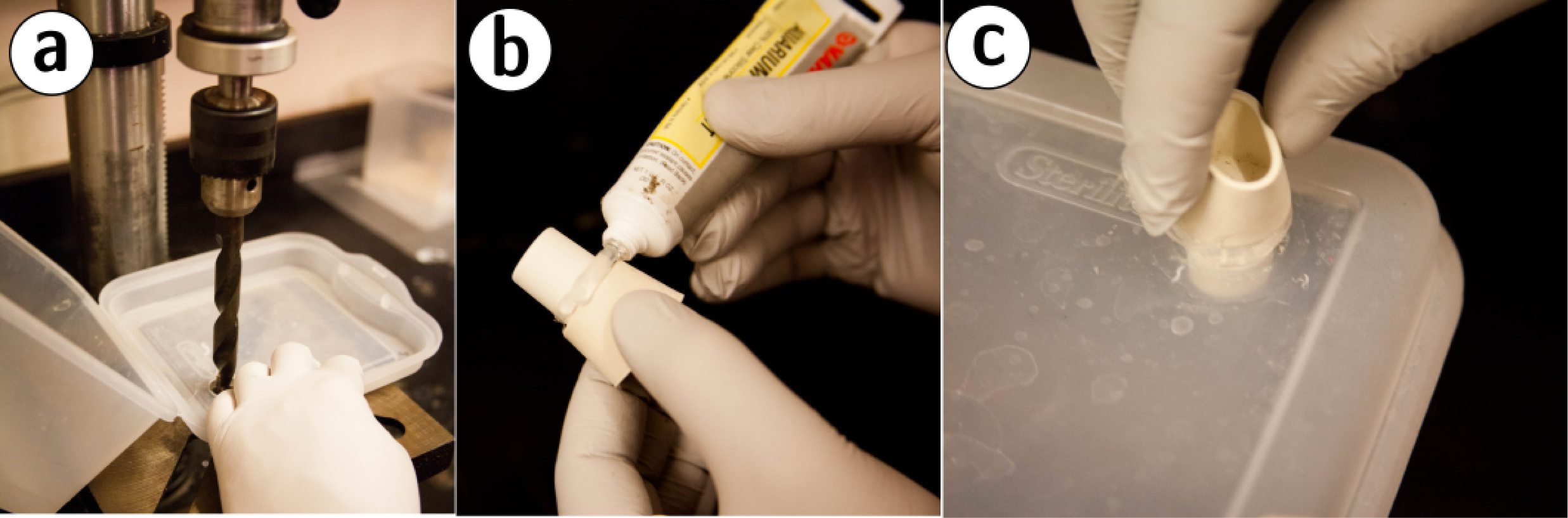

Supplement: S4 Fig — a.) 1” hole drilled into external container lid b.) Placement of port (TIF) [file pone.0155960.s008.tif]

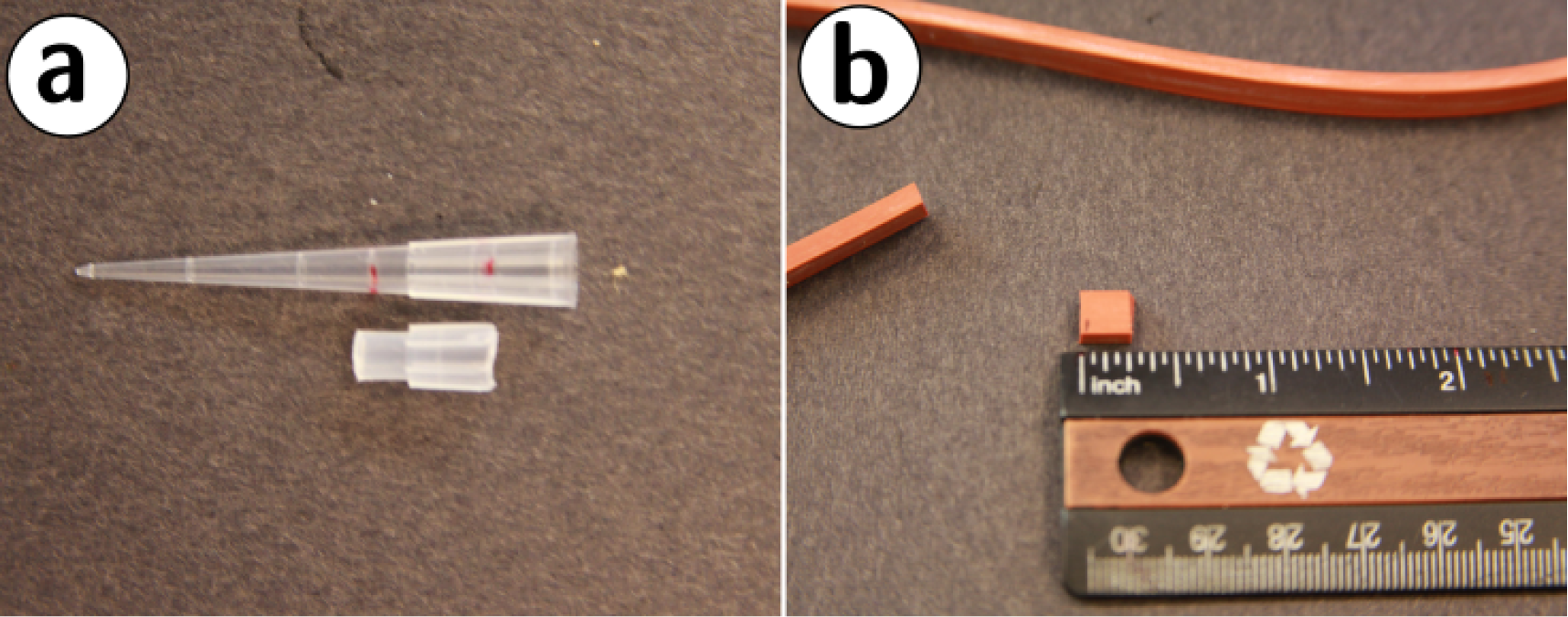

Supplement: S5 Fig — a.)Before cutting and after cutting pipette tip for agar plug b.) Silicone spacers (TIF) [file pone.0155960.s009.tif]

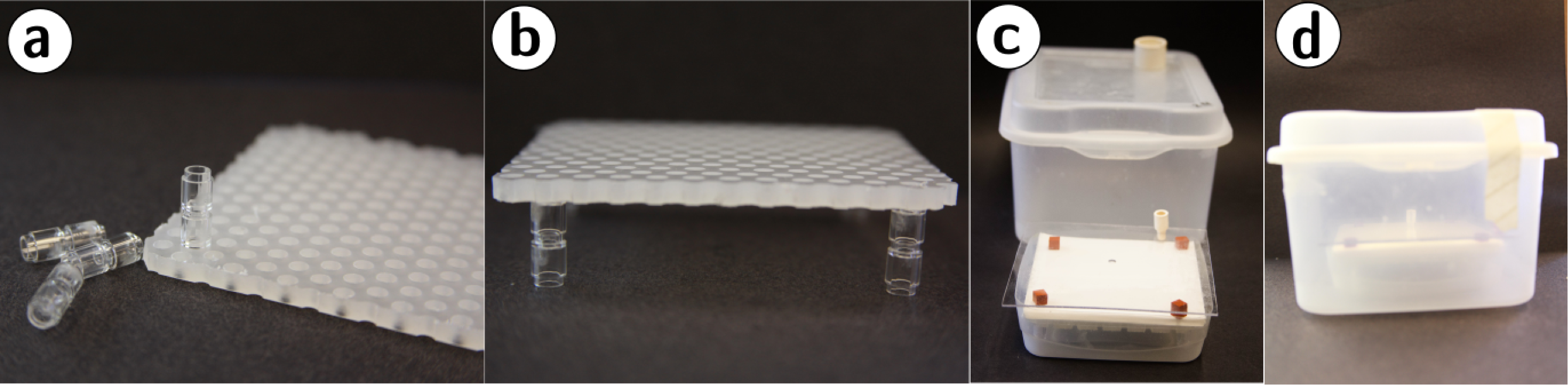

Supplement: S6 Fig — a.) Assembly of perforated LEGO® support b.) Finished LEGO® support c.) Preparing system for autoclaving d.) System ready for sterilization (TIF) [file pone.0155960.s010.tif]

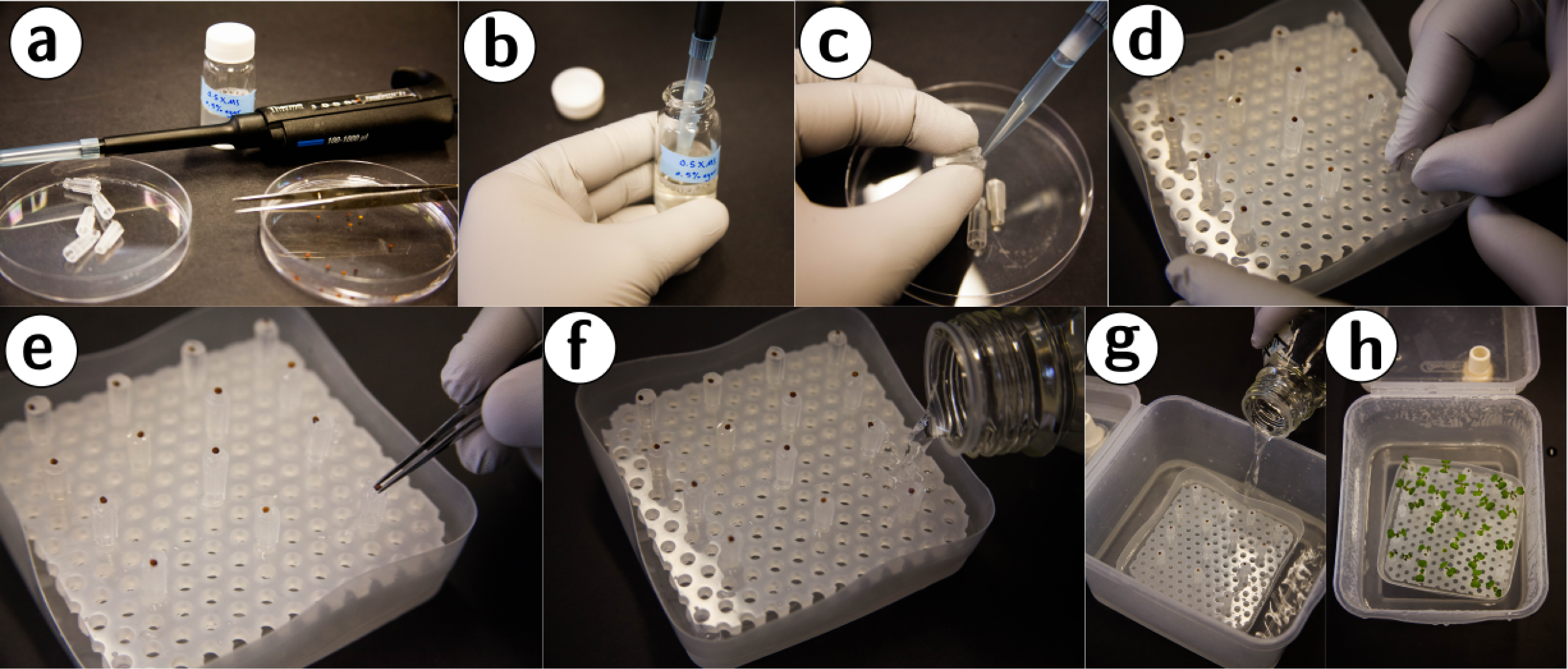

Supplement: S7 Fig — a-e.) Sterilized brassica seeds are placed in cured 0.5% agar with 0.5xMS after putting agar plugs into perforated plastic support f.) 0.5x MS is added to nutrient cup until contact is made between bottom of agar plug and MS solution g.) sterile water is added to height of inner nutrient cup level so nutrient cup does not have depletion of water h.) germination system with ~30 plants after 1 week from sowing seed. (TIF) [file pone.0155960.s011.tif]

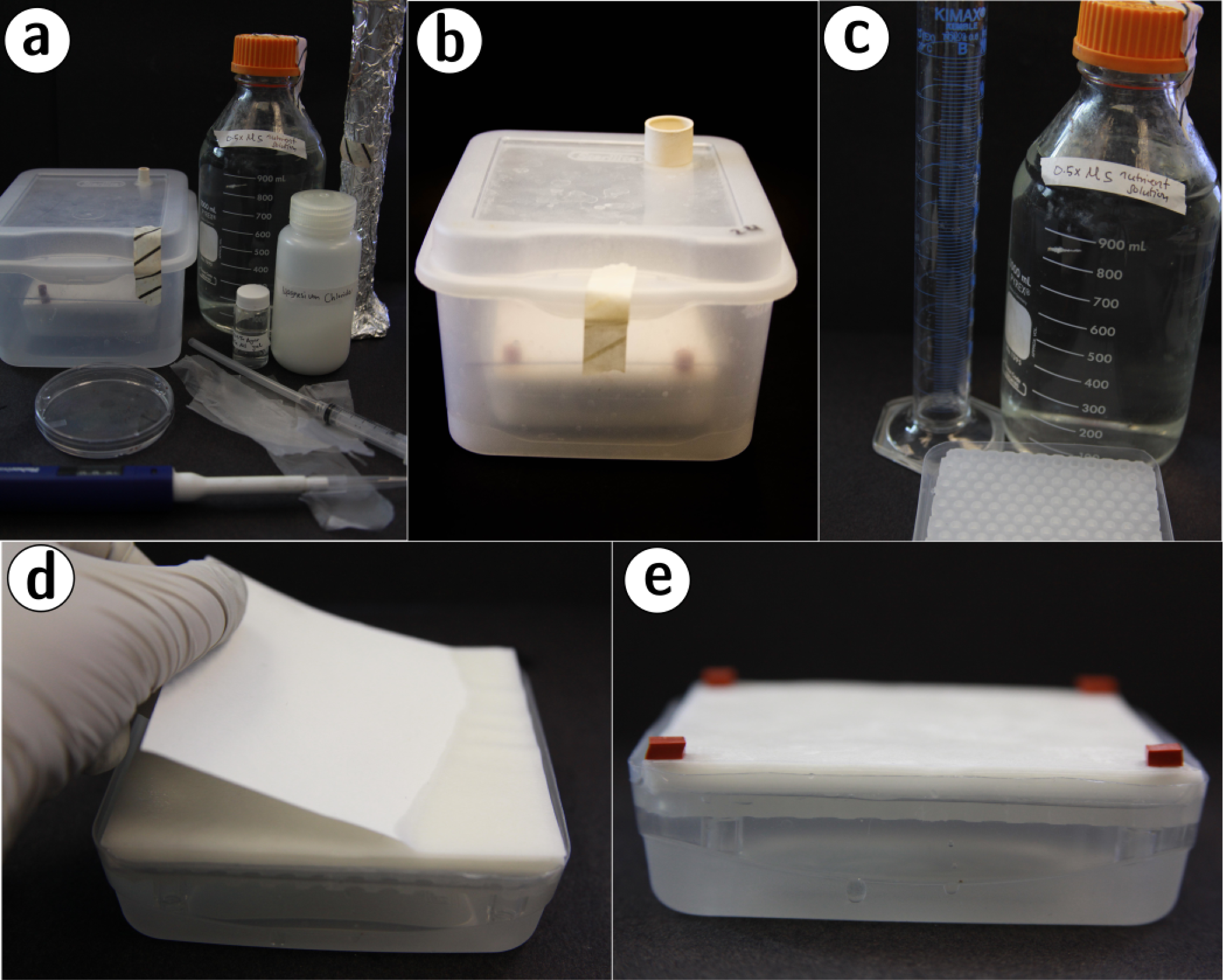

Supplement: S8 Fig — a-b.) materials used in system assembly c.) 0.5xMS added to nutrient cup with LEGO® brick support with perforated plastic sheet d.) paper pad is placed into cup and growth sheet is wicked across surface e.)rubber spacers are added onto each corner of paper surface (TIF) [file pone.0155960.s012.tif]

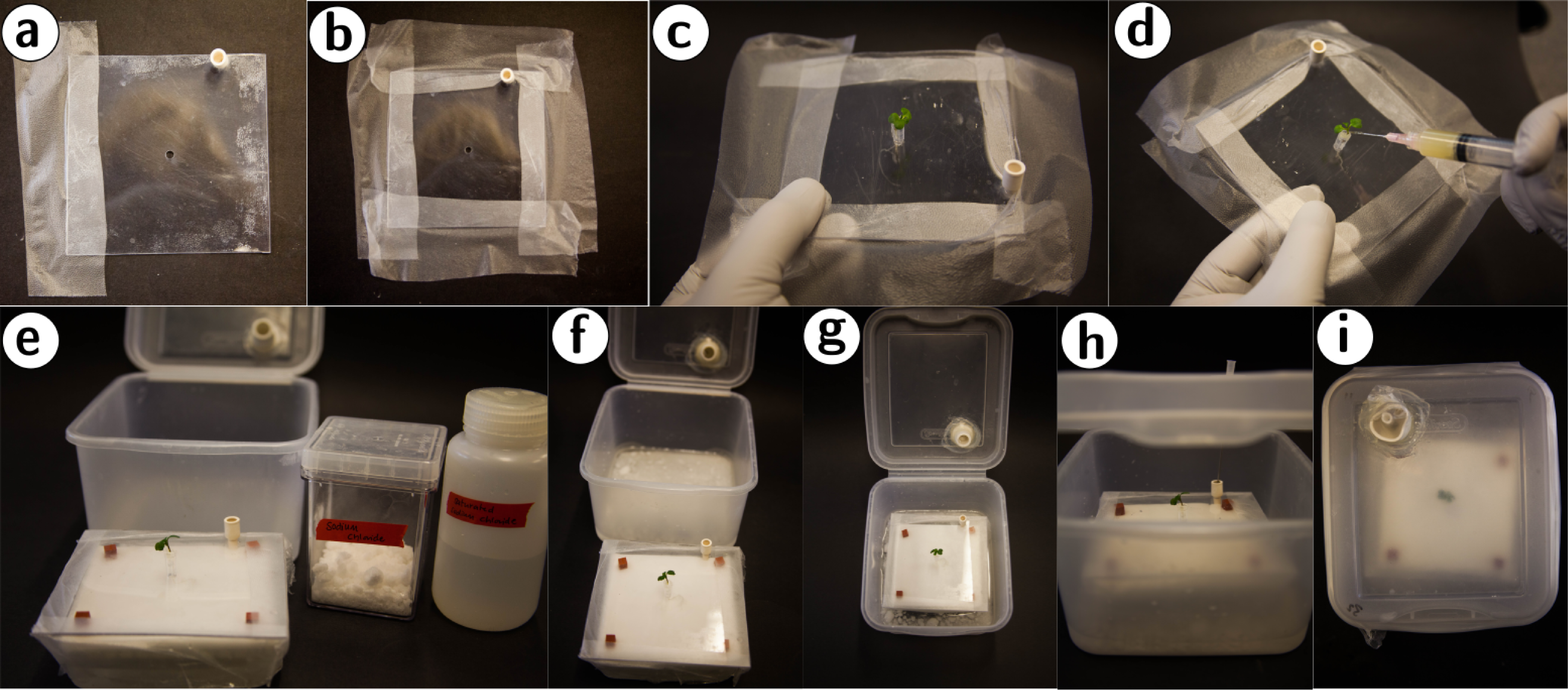

Supplement: S9 Fig — a-b.) plastic wrap is added to plastic seed support c.) plant from germination system is added by feeding root through central hole d.) sterile petroleum jelly is added to seal off agar plug thus preventing evaporation from plug. e-g.) plastic wrap is pressed against all side of nutrient cup like wrapping a gift and salt is added to control RH h.) a needle is added through both ports to allow access for sampling of cup and refilling of water i.) completed system with parafilm sealing around edges and over needle in port. (TIF) [file pone.0155960.s013.tif]

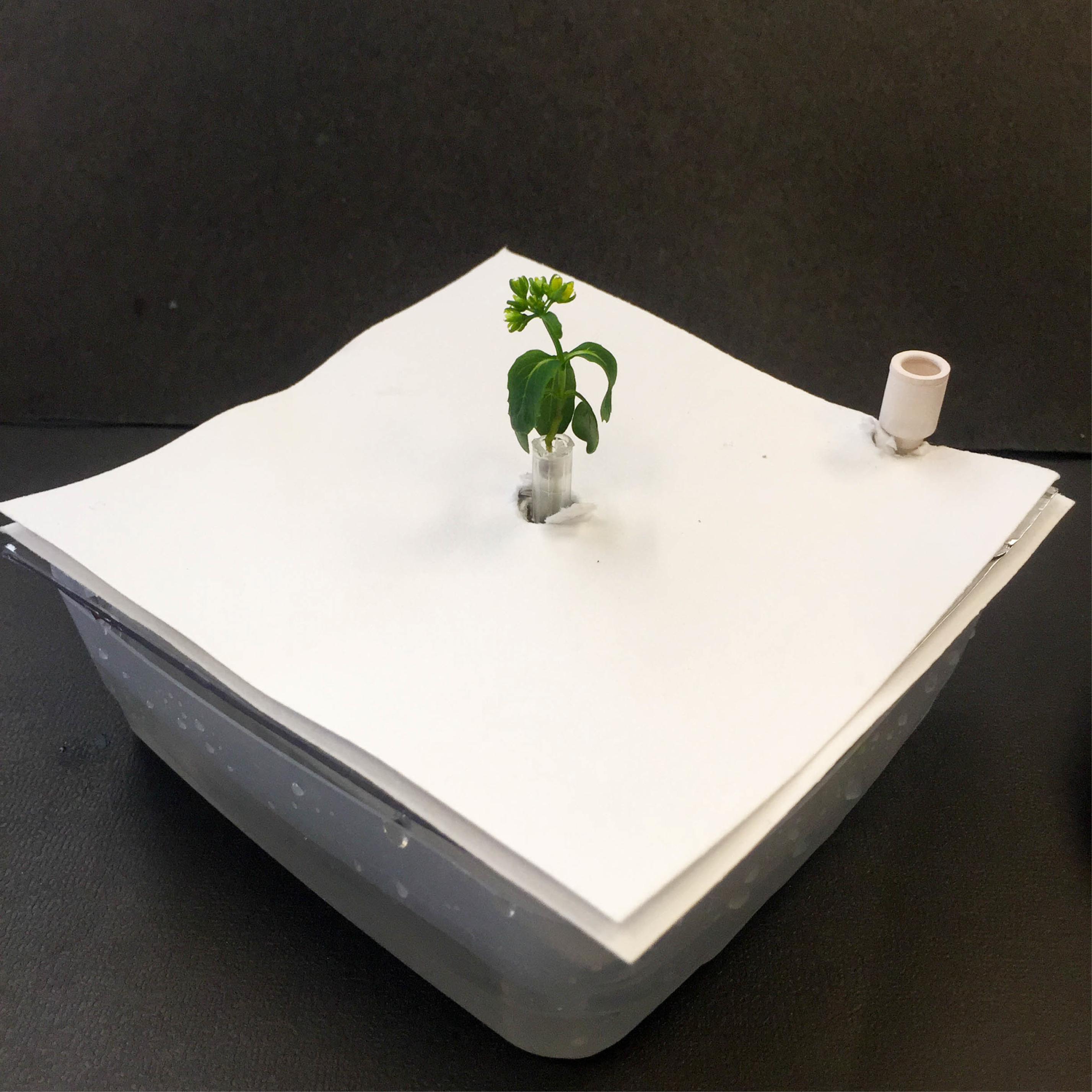

Supplement: S10 Fig — (TIF) [file pone.0155960.s014.tif]

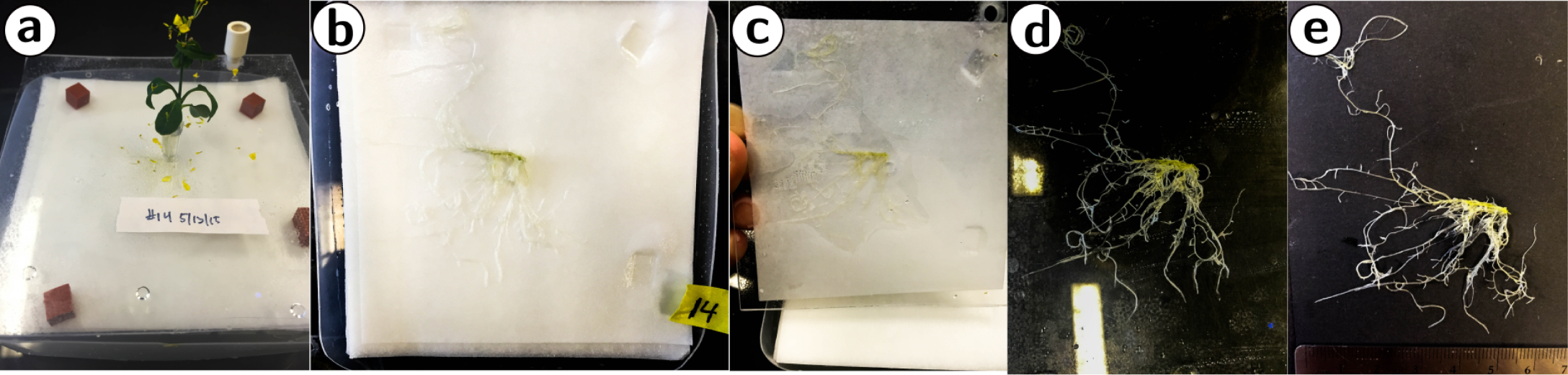

Supplement: S11 Fig — a-b.) the plastic wrap is removed from the system, the shoot is clipped from the root and the rubber spacers are removed. c.) the plastic sheet is used to invert the growth sheet with root d.) the growth paper is peeled away from the root unto instead the plastic sheet. e.) the plastic sheet is carefully removed to reveal the root geometry with contrasting background. (TIF) [file pone.0155960.s015.tif]

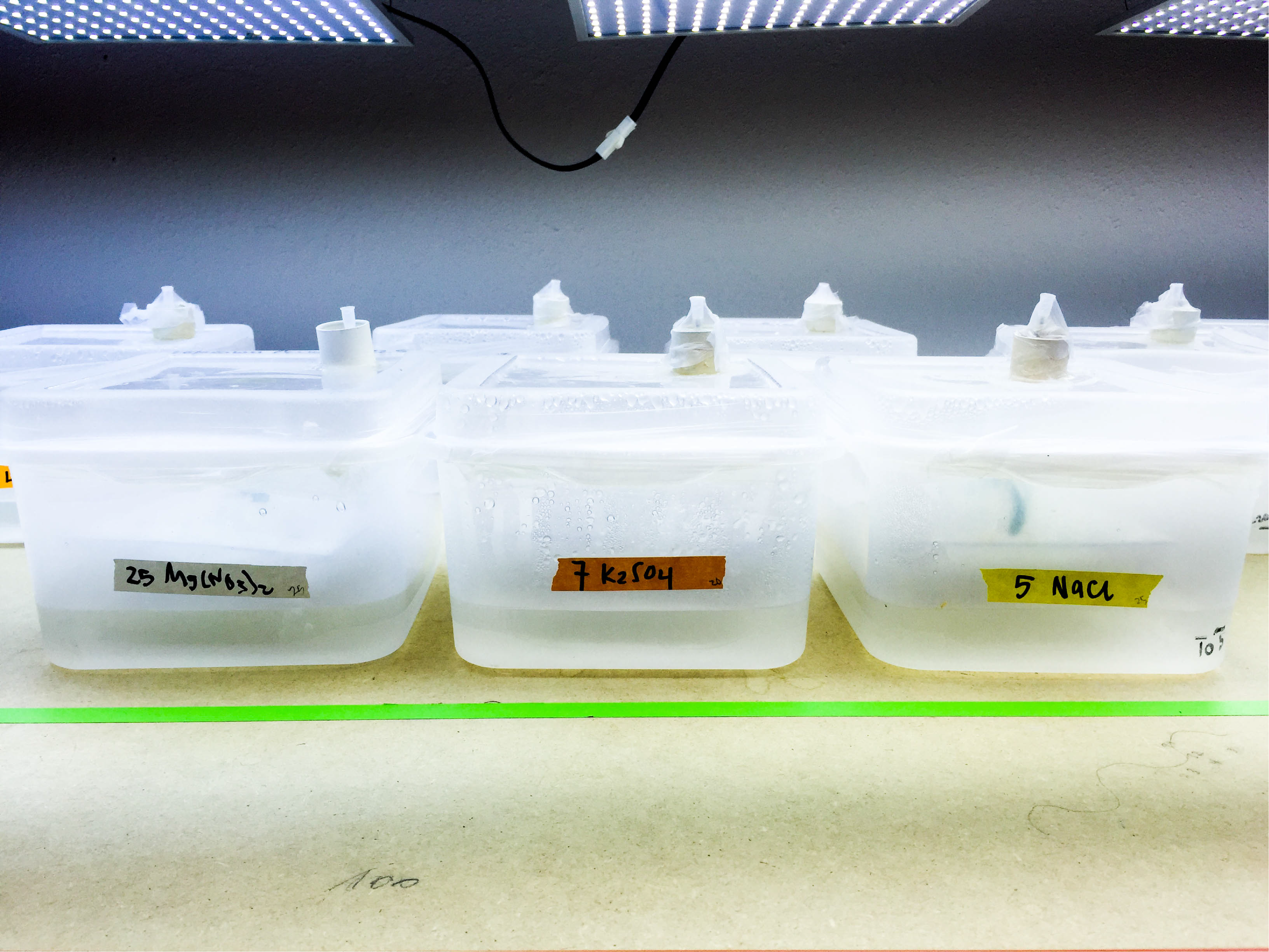

Supplement: S12 Fig — (TIF) [file pone.0155960.s016.tif]

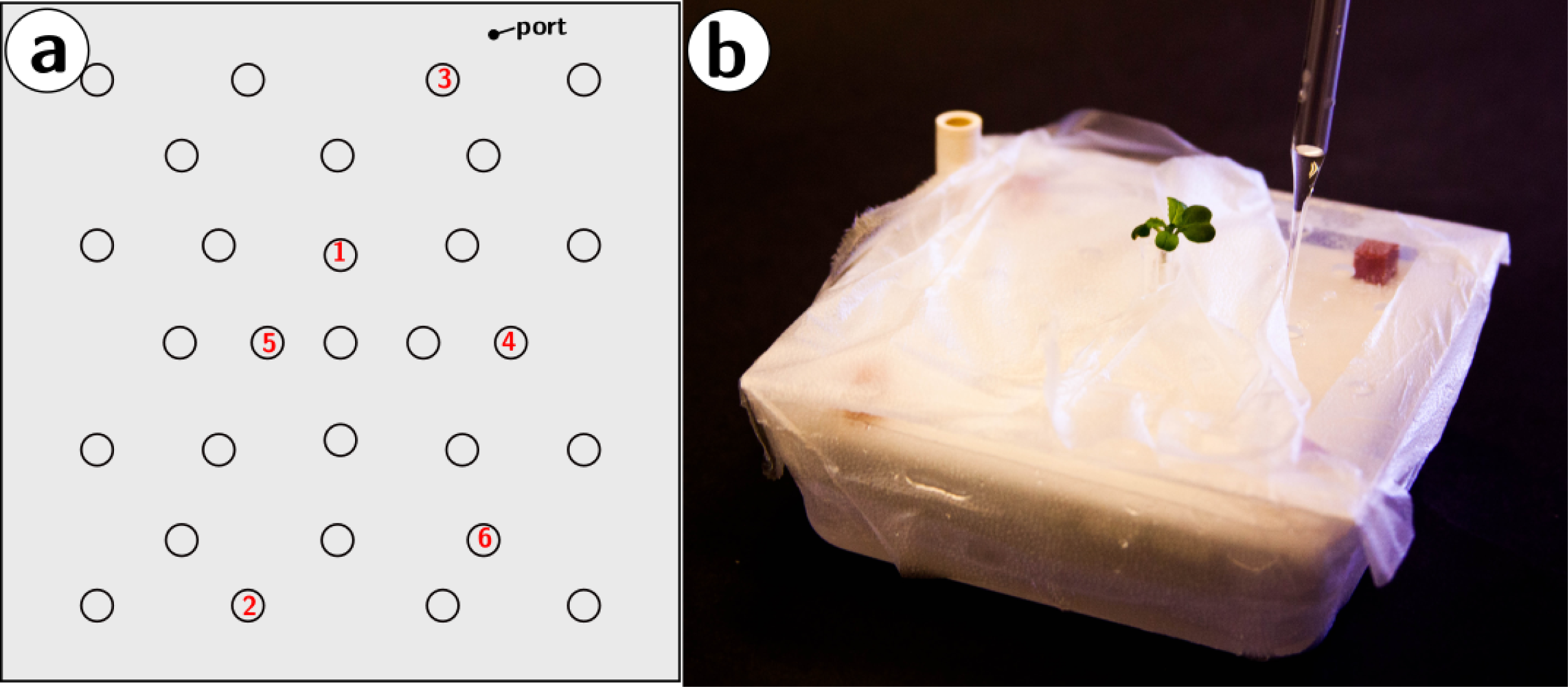

Supplement: S13 Fig — a.)plastic sheet with testing sites b.)glass capillary pipette testing procedure (TIF) [file pone.0155960.s017.tif]

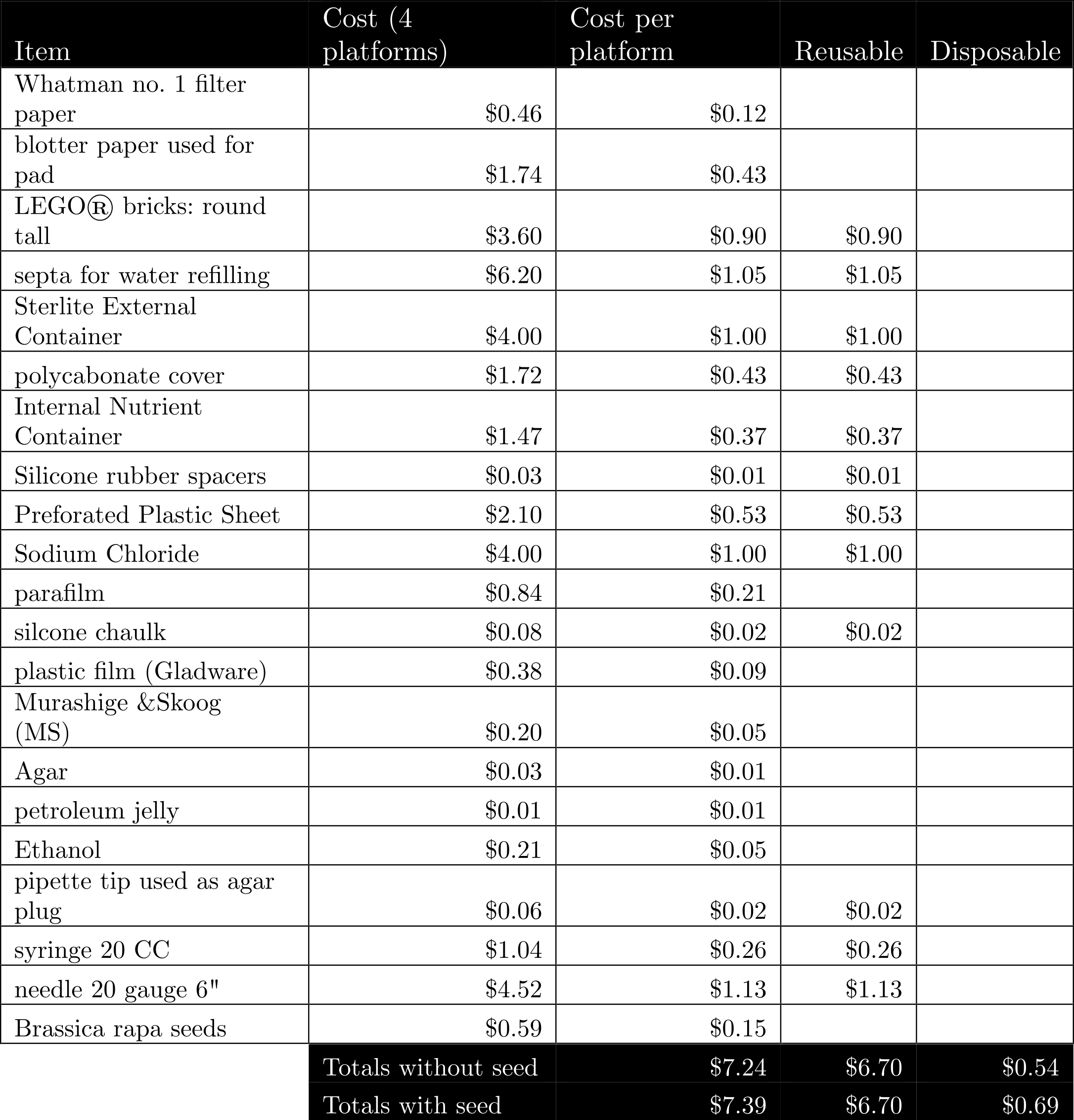

Supplement: S1 Table — (TIF) [file pone.0155960.s018.tif]
